# A review on synthesis of MOF-derived carbon composites: innovations in electrochemical, environmental and electrocatalytic technologies

**DOI:** 10.1039/d4ra05183a

**Published:** 2024-09-03

**Authors:** Sehar Shahzadi, Mariam Akhtar, Muhammad Arshad, Muhammad Hammad Ijaz, Muhammad Ramzan Saeed Ashraf Janjua

**Affiliations:** a Department of Chemistry, Government College University Faisalabad Faisalabad 38000 Pakistan Dr_Janjua2010@yahoo.com Janjua@gcuf.edu.pk +92 300 660 4948; b School of Chemistry, University of the Punjab, Quaid-i-Azam Campus Lahore 54590 Pakistan; c Department of Chemistry, University of Agriculture Faisalabad Faisalabad 38000 Pakistan

## Abstract

Carbon composites derived from Metal–Organic Frameworks (MOFs) have shown great promise as multipurpose materials for a range of electrochemical and environmental applications. Since carbon-based nanomaterials exhibit intriguing features, they have been widely exploited as catalysts or catalysts supports in the chemical industry or for energy or environmental applications. To improve the catalytic performance of carbon-based materials, high surface areas, variable porosity, and functionalization are thought to be essential. This study offers a thorough summary of the most recent developments in MOF-derived carbon composite synthesis techniques, emphasizing innovative approaches that improve the structural and functional characteristics of the materials. Their uses in electrochemical technologies, such as energy conversion and storage, and their function in environmental electrocatalysis for water splitting and pollutant degradation are also included in the debate. This review seeks to clarify the revolutionary effect of carbon composites formed from MOFs on sustainable technology solutions by analyzing current research trends and innovations, opening the door for further advancements in this rapidly evolving sector.

## Abstract 2

Carbon composites derived from Metal–Organic Frameworks (MOFs) have shown great promise as multipurpose materials for a range of electrochemical and environmental applications.

## Graphical abstract

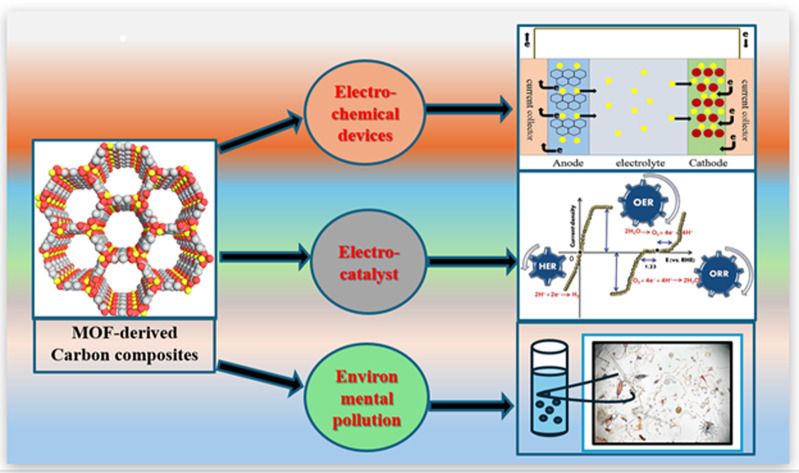

## 1 Introduction

Metal–organic frameworks (MOFs), with their fascinating properties including architectural stiffness, significant pore size, resilience to heat, chemical durability, crystalline frameworks, and limitless structural customizations, have become attractive options for different applications.^1^ MOFs are fascinating due to their unified pore structures, which offer flexibility for targeted motif decoration through physical or chemical means.^2^ They include a broad spectrum of chemical variability in metal clusters and permit organic linker functionalization without changing structural topologies.^3^ MOFs are very interesting because of these properties, secondary elements that are employed in the construction of hybrid frameworks include metal clusters and organic linkers. In particular, MOFs with hybrid functional structures provide cutting-edge platforms for sensing,^4^ adsorption,^5^ magnetic applications,^6,7^ and catalysis,^6^ among other uses. MOF chemistry is based on varied array of metal centered cluster that include nearly all metals of periodic table, such as s, p, d blocks metals and rare-earth elements.^8,9^ On the other hand, MOFs made of elements from the s-block, have gotten comparatively less attention because of least stability. Despite all this s-block MOFs are plentiful, economical, and have lower toxicity than transition metal-based MOFs. This makes them useful in a wider range of applications, especially in biological context. They are also beneficial for adsorption because of their low density.^10,11^ Among them, MOFs based on transition metals are especially well-known because of their unique properties, which include numerous oxidation states, different coordination geometries, and absorption of visible light. Clusters of Zr(iv),^12^ Fe(iii),^13^ Ti(iv),^14–16^ Cu(ii),^17^ Cr(iii),^18^ Al(iii)^19^*etc*, are often found in well-established MOF structures. Using carboxylate linkers in conjunction with high-valent metal cations is another method to improve the longevity of MOFs. Metal ions that are highly charged with increased charge densities can create robust coordination bonds when they use the same ligands and coordination conditions, creating a more durable structure.^12,20^ No doubt Zn^2+^ and Cu^2+^ based MOFs are more studied in last era but MIV-MOFs based on group 4 metals have gained the new era of research. This field was initiated with the first Zr-MOF appearance in 2008 (ref. 21) and Ti-MOF was discovered in 2009.^22^ However, their inception and importance has increased significantly, but the main reason of this, is their exceptional durability and vast applications. Earlier research has extensively reviewed the MOFs of metal ions such as Zr and Ti encompassing their synthetic methods, structural and intrinsic properties, and range of uses.^12,23,24^ Recent years have seen a notable increase in research interest in nanocomposites with desirable qualities originating from more than two phases due to their unusual combinations of properties and distinctive designs. Since they have the ability to get beyond the drawbacks of single-phases or micro-composites, they have been used in a number of fields, including bioengineering, sensing, catalysis, and renewable energy. The traditional method for creating composite nanomaterials involves applying a secondary phase by methods like hydrothermal, sol–gel, solution mixing, polymerization, and chemical vapor deposition to the interior or exterior surfaces of preexisting components.^25,26^ The majority of these processing techniques, however, still present difficulties with regard to stoichiometry, architecture, and chemical composition management.^27^ As a flexible method to develop nanostructured materials, including metal nanoparticles, porous carbons, and their composites, the thermal conversion MOFs of are simultaneously deserving of consideration.^28,29^

Furthermore, MOF-derived nanostructured functional materials have been beneficial in energy-related applications. MOFs can be readily transformed into inorganic functional materials through pyrolysis in an inert environment or chemical reactivity with suitable reagents, despite their inherent thermal and chemical instability. MOF precursors can be converted into metal-based compounds including carbon and their composites with various porous or hollow nanostructures, depending on the conversion process.^30^ Meticulous design and incorporation of MOFs and functional nanomaterials into innovative Multi compositional MOF-based nanocomposites could be utilized to manufacture complex nanostructured materials with designable shapes and compositions, in addition to employing simple MOF nanoparticles as precursors.^31,32^ Simple structural tuning of MOFs can be achieved by post-synthesis alteration as well as by choosing different metal cation and organic linker combinations. For instance, MOFs have significantly greater Brunauer–Emmett–Teller (BET) surface areas than zeolites and activated carbons, reaching up to 10 000 m^2^ g^−1^.^33^

Nevertheless, their applications are limited by morphologies, specific surface area and pore size control, which are problems with conventional synthesis methods of these materials like pyrolysis of organic molecules or biomass sources, elevated temperatures hydrothermal and solvothermal approaches, and vapor phase decomposition strategies.^34,35^ The MOF-derived carbon materials (MDCMs) have a number of benefits over other carbon materials, including consistent surface areas (almost 10 000 m^2^ g^−1^), good chemical stability in aqueous phases, and controlled porosity.^36^ These MDCMs combine the distinctive qualities of MOFs most notably, their exceptionally high specific surface area with the exceptional stability of carbon structures. Furthermore, by adjusting synthesis parameters, MDCMs' synthesis methods provide superior control over morphology, pore size, and surface area.^37^ This makes them more suitable for a variety of photocatalytic applications, such as the production of H_2_ and the reduction of organic pollutants and CO_2_.^38^

Carbon-based substances developed from MOFs often have more exposed active sites, a lower density, and have a simpler time being completely connected with the reaction medium than other types of carbon materials.^39^ Furthermore, the hollow carbon materials' internal cavity can effectively improve the active material's overall stability by increasing the active material's load and clearing the diffusion pathway while also acting as a buffer space for the active material's volume expansion for sustainable uses.^40^ Specifically, hollow porous carbon nanoparticles made from MOFs can have a range of morphologies, including sphere, cube, dodecahedron, and tube structures, and their diameters for the particles and cavities vary from tens to hundreds of nanometers.^41,42^

The number of pollutants released into the environment as a result of industrial and agricultural development is increasing, and this has a detrimental effect on human health. Effective methods of dealing with these pollutants include the catalytic method, which breaks down pollutants into harmless degradation products. The key is to design and develop highly efficient, non-toxic, and chemically stable catalysts that are easily synthesised in a variety of sizes and shapes in order to obtain controllable pore structure and abundant active sites.^43^

Our goal in this review is to provide a concise overview of the synthesis strategies of MOF-derived carbon composites, including their chemical makeup and frameworks, for usage in different applications (energy storage devices, catalysis, environmental). In particular, methods for altering the composition and morphology are discussed. Finally, a peek forward at the current developments and unresolved issues with MOF-derived carbon composite research is provided.

### 1.1 MOFs

MOFs are porous crystalline structures that are created by metal ions inside the lattice that are joined by organic linker molecules. The direction of bond between metal and linker inside lattice determines the voids and empty spaces. Because these bonds are more resilient than covalent bonds and have a moderate energy level, the stability of the framework depends on them. Metal nodes with unbound coordination sites, functional linkers, or guests tucked away in the voids can give rise to active sites in MOFs. Structural anomalies also serve as catalyst sites. A number of characteristics, including the capability to manufacture MOFs with different metals, large surface and pore sizes, robust lattice structures, unsaturated metal sites that are specific, and simple synthesis and design procedures, have made the MOFs as promising solid catalyst materials.^44^ MOFs have become exceptional materials during the past 20 years because of their outstanding achievements across multiple domains including catalysis, storage, and separation. Monovalent (Cu^+^, Ag^+^) or divalent (Mg^2+^, Mn^2+^, Co^2+^, Zn^2+^, Ni^2+^, Fe^2+^, Cu^2+^, Cd^2+^), trivalent (Al^3+^, Sc^3+^, V^3+^ Cr^3+^, In^3+^, Fe^3+^, Ga^3+^), or tetravalent (Zr^4+^, Ti^4+^, Hf^4+^) metal cations are often the origin of the inorganic nodes of MOFs. When MOF investigation first started, metals with +2 oxidation states such as Cu^2+^ and Zn^2+^ were common selections.^45^ Nevertheless, despite their advantages, MOFs made of these divalent metals have limited uses since they are unstable in adverse environments. Maintaining MOF structural integrity is essential to maintain their intended features and capabilities throughout a range of applications. Water and moisture are typically found in industrial operations (*i.e.* catalysis) need coordination of anions or stability against aqueous acid/base conditions. Regrettably, the vulnerability of several MOFs to deterioration in aqueous or hostile surroundings has substantially impeded their wider use and economic feasibility.^12,46^ As such, the focus of current researcher efforts has turned to creating stronger framework structures.^47^

### 1.2 Main group metal-based MOFs

d-block elements and in more recent development f-block metal cations are the main constituents of the majority of MOFs. s-block metals, alternatively, received less attention. This may be due to the widespread belief, similar to that of lanthanide elements, that they are less appropriate as core metal ions for different kinds of MOFs. Some s-block metals-based MOFs are [H_2_N(CH_3_)_2_]_2_Ca_7_(BTB)_5_(H_2_O)_8_(DMF)_4_·4H_2_O (1), [H_2_N(CH_3_)_2_]_2_Sr_5_(H_2_O)_6_(BTB)_4_ (2), and [H_2_N(CH_3_)_2_]Ba(H_2_O)(BTB) (3). Each of them displays 3D structure and diverse secondary building components. Compound 1 features zigzag chain of Ca–O–Ca in linear arrangement; compound 2 contain pentameric cluster of Sr_5_O_28_; and compound 3 include chains of Ba–O–Ba also arranged in linear dimension.^10^ While MOFs derived from s-block metals have received less attention, but they offer a range of intriguing properties.

Some s-block metal ions with substantial biological significance are Na^+^, K^+^, Mg^2+^, Ca^2+^, and Ba^2+^. They are desirable candidates for the designing biocompatible MOFs due to their biocompatibility.^48,49^ For instance, barium (Ba^2+^) is frequently used in medical imaging as an X-ray contrast agent.^50^ Consequently, there is a lot of potential for medical applications when these s-block metal ions are combined with biocompatible linkers.^51^

As s block metal ions, such magnesium (Mg) or lithium (Li) are less dense, it can be used to design MOFs with large surface areas and minimal densities, which will improve their capacity to absorb gases. For instance, by utilizing lithium Li^+^ to modify MOFs gravimetrically researchers have designed MOFs with encouraging gas sorption capabilities. Additionally, the ability of magnesium-based MOFs to selectively collect and separate CO_2_ has been thoroughly investigated.^10^

Apart from Open Metal Sites (OMS), infinite chain SBUs are a common characteristic of s-block MOFs structures, functioning as strong Lewis acidic sites. These diverse SBUs are essential for improving the ability to absorb pollutants and promoting organic transformations. This is primarily because these SBUs have dense catalytic active sites.^10,52^

### 1.3 Li based MOFs

**Fig. 1:**
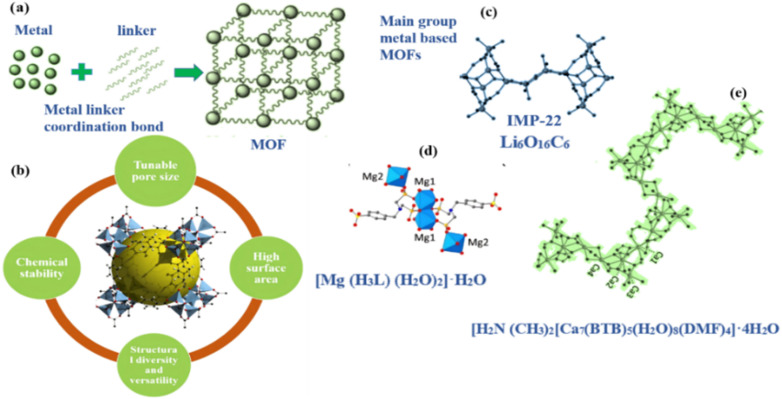
Illustrating (a) metal and linker bonding to form MOF. (b) Different properties of MOFs. (c) Lithium metal-based MOF. (d and e) Magnesium and calcium metal-based MOF.

Lithium (Li) is unique among the alkali metals since it is the lightweight element and usually forms crystals with tetrahedral shape shown in Fig. 1. A good example of this is observed with the combination Li_4_(L1)_2_(H_2_O)(DMF)_2_, sometimes identified as IMP-22 in finding of Pugh *et al.*^53^ For this scenario, DMF stands for *N*,*N*′-dimethylformamide, while L1 stands for 4,4′-dimethylsilanediyl) dibenzoic acid. The SBUs in lithium-based (MOF) frameworks are divided into two separate components. An 8-membered ring and its core (Li_2_O_4_C_2_) is joined to Li_2_O_2_ (a ring of 4 members) to form the tricyclic structure of the first subunit. The second component is a Li_6_O_16_C_6_ cluster that is made up of two coordinated solvent molecules (DMF) and six carboxylate functions that connect to six lithium cations. Although lithium-based MOFs comprise SBUs characterized by altering rings with four and eight member each.^54^ IMP-22 represent the pioneering lithium MOF to feature the SBUs with three interconnected ring. Li polyhedra within this framework are linked to –COO^−^ groups.^10^

### 1.4 Mg based MOFs

Wöhlbrandt and colleagues synthesized and characterized the [Mg(H_5_L_4_)(H_2_O)_2_]·H_2_O whereby H_5_L_4_ stands for 4-{[bis(phosphono-methyl)amino]methyl} benzenesulfonic acid.^55^ Every Mg^2+^ ion in the structure of [Mg(H_5_L_4_)(H_2_O)_2_]·H_2_O is bounded to six atoms of oxygen. Two atoms of oxygen come from coordinated water molecules, while the remaining four oxygen atoms originate from three H_3_L^2−^ ligands that are attached to Mg^2+^ ion *via* phosphonate groups. Together, these connections created an MgO_6_ polyhedron. Three Mg^2+^ ions are connected to each linker, and the water molecule in third position between the sequential alignment is where hydrogen bonding takes place.^10^

### 1.5 Ca based MOFs

In alkaline earth metal-based MOFs, calcium-based clusters typically crystallize into infinite chains with varying coordination numbers. A calcium-based MOF, for example, [H_2_N(CH_3_)_2_[Ca_7_(BTB)_5_(H_2_O)_8_(DMF)_4_]·4H_2_O was reported by Asha *et al.*^10^ Calcium ions (Ca^2+^) show varying number of connections: Ca_1_ forms CaO_7_ polyhedron by linking to seven atoms of oxygen, whereas Ca_2_ bonds to nine oxygen atoms to form a CaO_9_ polyhedron. In addition, Ca_3_ and Ca_4_ ions adopt an irregular octahedral form. When BTB ligands attach themselves with calcium atoms, a three-dimensional structure is formed in which the calcium ions are connected by Ca–O–Ca connections to form endless zigzag patterns shown in Fig. 1.

### 1.6 Transition metal-based MOFs

Adding carboxylate linkers and high-valent metal cations to MOFs is one way to improve their stability.^20^ Metal cations with higher positive charges can forge more robust coordination bonds because of their greater charge density and a more durable structure by using identical ligands and coordination surroundings. When metal cations of group 4 are combined to –COO^−^ based ligands, they are expected to form robust MOFs since they typically have an oxidation state of +4. Moreover, due to boosted metal ligand interactions, these metal cations with +4 charge require additional ligands to offset charge. As such, the inorganic vertices are usually well connected, which reinforces the system's durability by deterring any onslaughts from visitors *i.e.* water molecules.^45^ In MOF structures, Group 3 metal cations usually have an oxidation state of +3, whereas Group 4 metal cations have an oxidation state of +4. As a result, group 4, form far stronger coordination connections with carboxylates. One of distinguishing characteristics of MOFs including metals from group 3 and 4 is their capacity to form phases containing M_6_O_8_ clusters (where M = Y, Ln, Zr, and Hf), irrespective of atomic properties, charges or sizes. The notable series of M_6_ based MOFs, compounds like UiO-66 from UiO series with the face centered cubic structure, demonstrates this adaptability.^56,57^

### 1.7 Zr-based MOFs

MOFs of zirconium metal are highly regarded in the realm of real-world applications owing to their outstanding durability and strength, diverse range of structural types, and fascinating characteristics and functionalities.^12^ MOFs of Zr have acquired significant attention owing to their remarkable resilience to increased defect concentrations without experiencing significant loss of stability or crystallinity. This is associated with the robust interconnectivity of Zr_6_ clusters and their potential to decrease their maximum connections.^58^

**Fig. 2:**
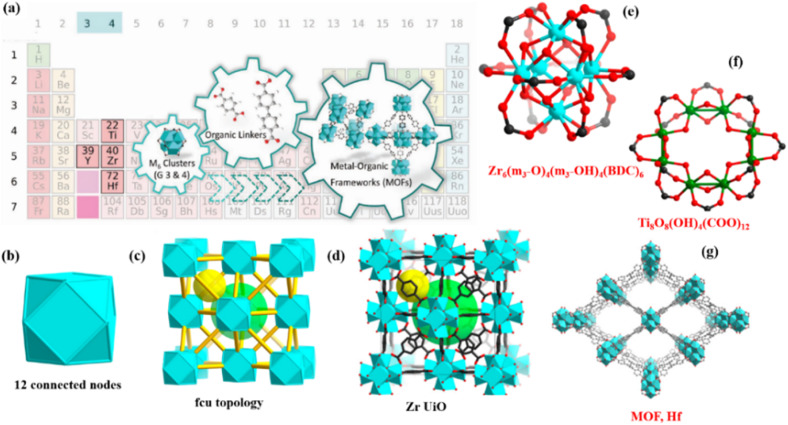
Illustrating (a) group 4 metals in periodic table. (b) 12 connected nodes for group 4 metal clusters. (c) Face centered cubic topology for MOFs showing arrangements of nodes. (d and e) Zr UiO MOF. (f and g) Ti and Hf MOF.

Cavka *et al.*,^21^ discovered the Zr_6_(m_3_-O)_4_(m_3_-OH)_4_(BDC)_6_ coordinated with Zr_6_ cluster four (m_3_-O), four (m_3_-OH) and 12(CO_2_) clusters. The Zr_6_ cluster is unique in that it may change the number of connections while preserving the sturdy [Zr_6_(μ_3_-O)_4_(μ_3_-OH)_4_] central structure shown in Fig. 2. Unprecedented stability is shown by the structure of UiO-66, particularly hydrothermal stability that surpasses the majority of known MOFs.^59,60^

### 1.8 Ti-based MOFs

MOFs with titanium clusters have many advantages in comparison to Zr-MOFs, including excellent stability, strong photocatalytic activity, and low toxicity. MIL-125, the pioneering Ti-MOF, was first described by Serre and colleagues in 2009. It is made up of ditopic BDC linkers and [Ti_8_O_8_(OH)_4_(COO)_12_] clusters which is 12 connected and has a fcu structure similar to the well-known Zr based MOF such as UiO-66, MIL-125 exhibits persistent porosity shown in Fig. 2. However, due to the extremely reactive character of the Ti precursors it was used to create TiO_2_. Since 2018 there has been limited number of Ti based MOFs. The recently discovered MOFs include a variety of titanium building blocks, including as Ti_3_ clusters, Ti_8_O_8_ grouping, Ti_12_O_15_ assemblies, sheets and chains of Ti–O bonds and isolated TiO_6_ metal cluster.^57^

### 1.9 Hf based MOFs

As Hf-MOFs are commonly presumed that they have comparable physical characteristics and coordination structures to their Zr counterparts.^61,62^ When synthesized under similar circumstances, the fabrication of Hf-MOFs is usually compared with Zr-MOFs, simply substituting Zr reagents with hafnium ones. The strong mechanical, thermal, and chemical stability seen in frameworks such as UiO-66 is preserved by this replacement.^63,64^ Moreover, Hf SBUs have more potent acidic sites compared to Zr-SBUs because Hf–O exhibits dissociation enthalpy of bond 802 kJ mol^−1^ whereas Zr–O bond shows (776 kJ mol^−1^).

## 2 Strategies for synthesizing MOF-derived carbon nanomaterial

There are many different ways to prepare carbon materials.^65^ These include carbonizing organic precursors directly, carbonizing carbon physically or chemically, utilizing zeolites and mesoporous silica as template materials, using high-temperature solvothermal and hydrothermal methods, using electrical arc methods, and using chemical vapor decomposition (CVD) methods.^66^ Because of its simplicity and versatility, the most common method for creating nanoporous carbons (NPCs) is direct carbonization from organic precursors.^67^ These NPC materials do, however, have some shortcomings that will severely restrict their uses, such as low surface areas, chaotic architectures, and uneven sizes.^68^ As research has advanced, scientists have discovered that carbon materials made from MOFs have the potential to transcend these constraints.^69^

Additionally, Wang *et al.* showed a unique method for converting MOFs without any catalytic activity into carbon-based materials and showed excellent stability and storage capacity. This method was created using MOF-guest precursors, and because of the special guest impregnation, all the porous MOFs that can take the place of the guests may also be utilized as the precursors, expanding the method's possible application.^70^

**Fig. 3:**
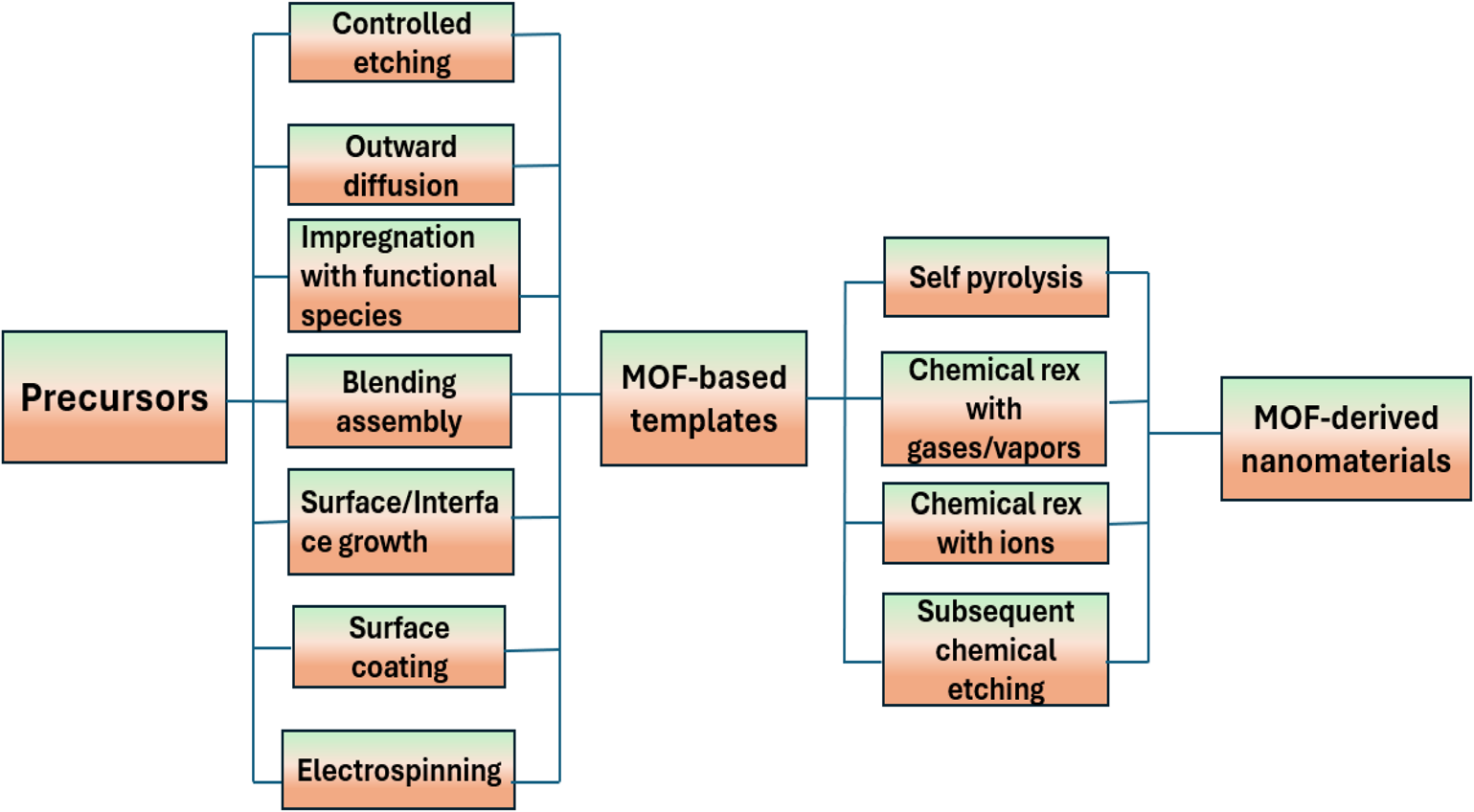
A general overview of synthesizing strategies for MOF based precursors and their derived nanomaterials.

The synthesis of nanomaterials produced from MOFs with precisely regulated structures and compositions provides an opportunity to examine and modify their electrochemical characteristics. Several techniques for synthesizing MOF based precursors and converting them into functional materials are discussed in Fig. 3.^71^ Here, we go over how to manipulate two processes to generate nanostructured materials from MOFs:

(i) Creating precursors based on MOFs and

(ii) Converting those precursors into the appropriate functional materials.

When metal ions and organic ligands are combined under suitable reaction circumstances, solution-based techniques may typically produce MOF particles with tunable sizes, shapes, and compositions. Then, to create various nanostructured MOF-derived materials, self-pyrolysis in an inert atmosphere or chemical reaction with various reagents, like reactive gases, vapors, and ions in solutions, are usually used. Conventional MOF particles have frequently been employed as precursors in earlier research. However, the majority of recent research shows that MOF nanostructures and MOF-based composite precursors would present new possibilities for the synthesis of highly complex nanostructured materials in terms of composition and architecture.^30,72^

## 3 1st strategy

### 3.1 Pyrolysis of MOFs for synthesizing porous carbon

**Fig. 4:**
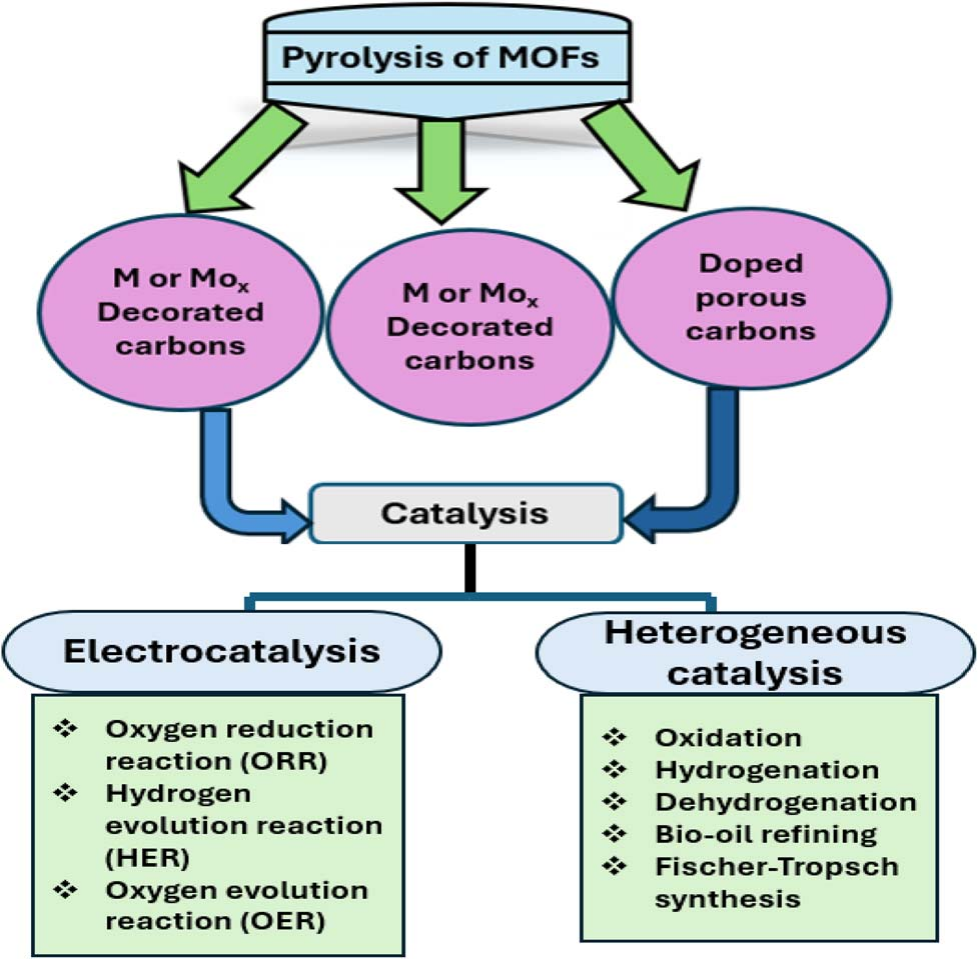
Several carbon-based nanomaterials derived from pyrolysis of MOFs.

Pyrolysis is the method that has been explored the most in relation to all the many techniques that have been devised to synthesised carbon compounds as shown in Fig. 4. The abundant organic ligands function as precursors in the MOF pyrolysis process, generating a range of carbon compounds with unique structures and forms.^73^ MOFs can be carbonized most typically *via* pyrolysis in a regulated environment and temperature, which yields derived carbons with distinct nanostructures. In significant part, the preparation technique and chemistry of the parent MOFs determine the structure of carbons generated from MOFs. The shape, pore structure, and surface chemistry of MOF-derived carbons are further influenced by the choice and loading of organic ligands, pyrolysis temperature, and post-modification. Performance is determined by the structure of carbons. Numerous investigations have demonstrated that MOF-derived carbons have promising futures in the catalysis industry.^74^

**Fig. 5:**
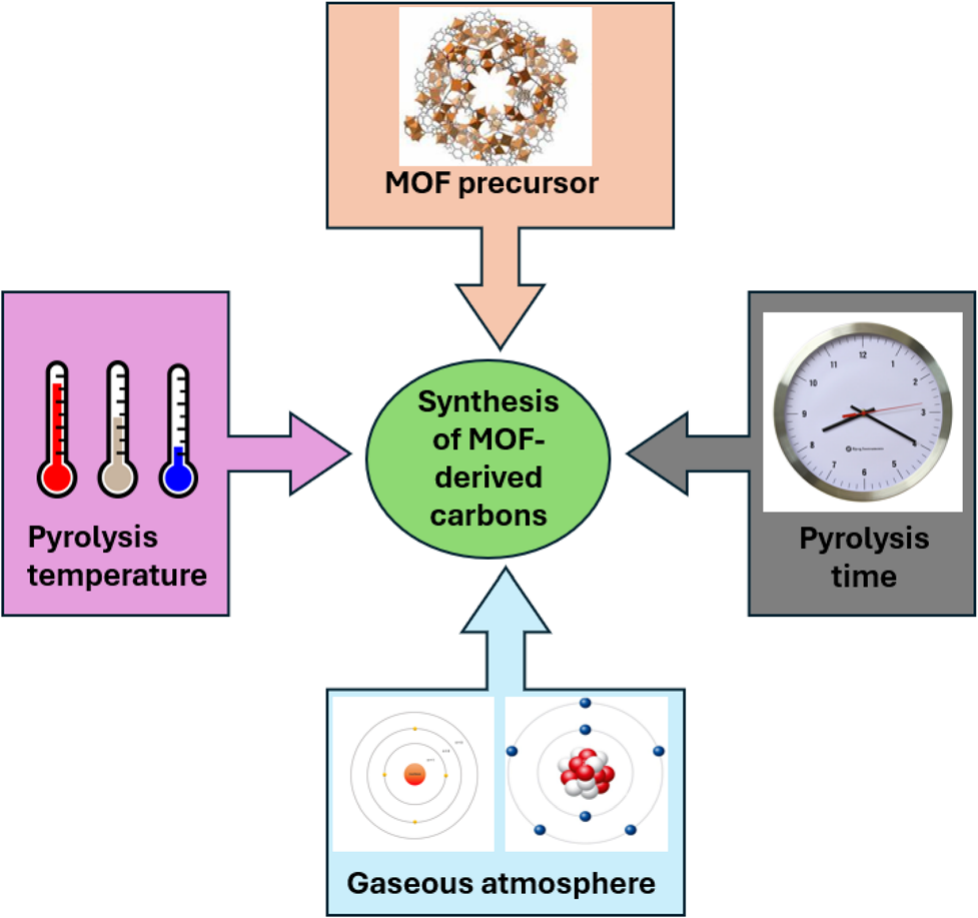
Major parameters that effect the synthesis of MOF derived carbon nanomaterials.

However, as shown in Fig. 5 and explained in the points that follow the synthesis of MOF-derived carbon nanomaterials are dependent upon a few factors, (i) the MOF precursors, where the carbon compounds that are produced typically maintain the morphologies of the MOF precursors that were first used. Selecting the appropriate MOF precursor can also alter the resulting material's textural characteristics.^75^

(ii) The pyrolysis time, as changing the heating duration can have a significant impact on the shape, size, and chemical makeup of the carbon nanomaterials that are produced.^76^

(iii) The gaseous environment, in which various pyrolysis gas atmospheres, such as oxidizing gases (air, O_2_, CO_2_, and H_2_O), and reducing gases (H_2_, NH_3_, and H_2_S), inert gases (Ar, He, and N_2_), can be used to generate hydrophobic and/or hydrophilic functionalized carbon nanomaterials.^77^

(iv) By adjusting the pyrolysis temperature one can alter the atomic ratios and crystalline phases of metal oxides (FeO_*x*_, TiO_*x*_, CuO_*x*_, *etc.*) contained in the carbon nanomaterials generated from MOF.^66,78^

MDCMs are mainly governed by 2 techniques, (1) MOFs *via* direct pyrolysis and (2) mof pyrolysis *via* guest encapsulating species.

### 3.2 MOFs *via* direct pyrolysis

**Fig. 6:**
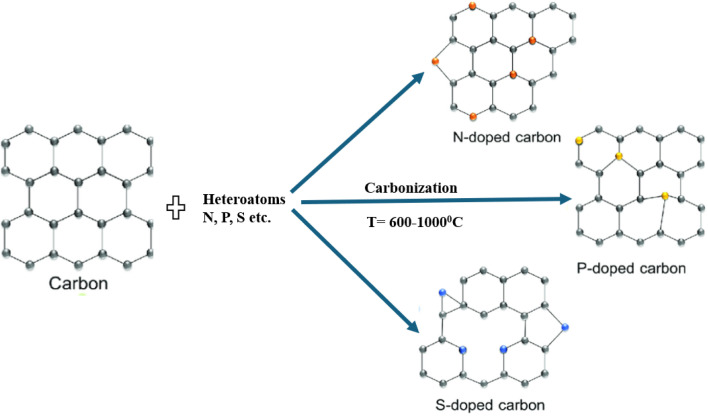
Doping of porous carbon with heteroatoms like S, P, and N.

In order to create porous carbon structures with predetermined properties, the MOF precursors or templates are usually carbonized in an inert environment (such as Ar or N_2_). This is followed by the leaching of metal species. By directly pyrolyzing ZIF-8, Jiang *et al.* produced porous carbons with a surface area as high as 3067 m^2^ g^−1^, a groundbreaking discovery. Furfuryl alcohol has been found to increase the surface area (3405 m^2^ g^−1^) of the resulting porous carbons upon introduction and polymerization in ZIF-8 MOF. Then, using a straightforward and easy pyrolysis method, Hu *et al.* created nanoporous carbons with an extremely large surface area (5500 m^2^ g^−1^). Importantly, as Fig. 6 illustrates, porous carbon doped with different heteroatoms (N, P, S, *etc.*) can also be created by directly pyrolyzing MOFs containing heteroatom and functional groups (–NH_2_, –SO_3_H, *etc.*).

### 3.3 MOF pyrolysis *via* guest encapsulating species

The introduction of guest species into MOF pores followed by pyrolysis is another well-liked synthesis technique to develop MOF-derived porous carbon nanoparticles. When guest species are introduced into MOF pores and then pyrolyzed, it is possible to generate porous carbons with more active sites that can be used for a variety of gas adsorption, catalytic, and energy storage applications. Pyrolysis of MOFs may result in a limited number of active sites. In order to create porous carbons with a large surface area (2872 m^2^ g^−1^), Xu's group used the pyrolysis of furfuryl alcohol-incorporated MOF-5 as a suitable precursor. When MOF-5 was pyrolyzed in an inert atmosphere, carbon was added to the resultant ZnO species, which was then reduced to produce evaporative Zn at temperatures above 700 °C and highly porous carbon.

## 4 2nd strategy

### 4.1 Carbonization of MOFs

**Fig. 7:**
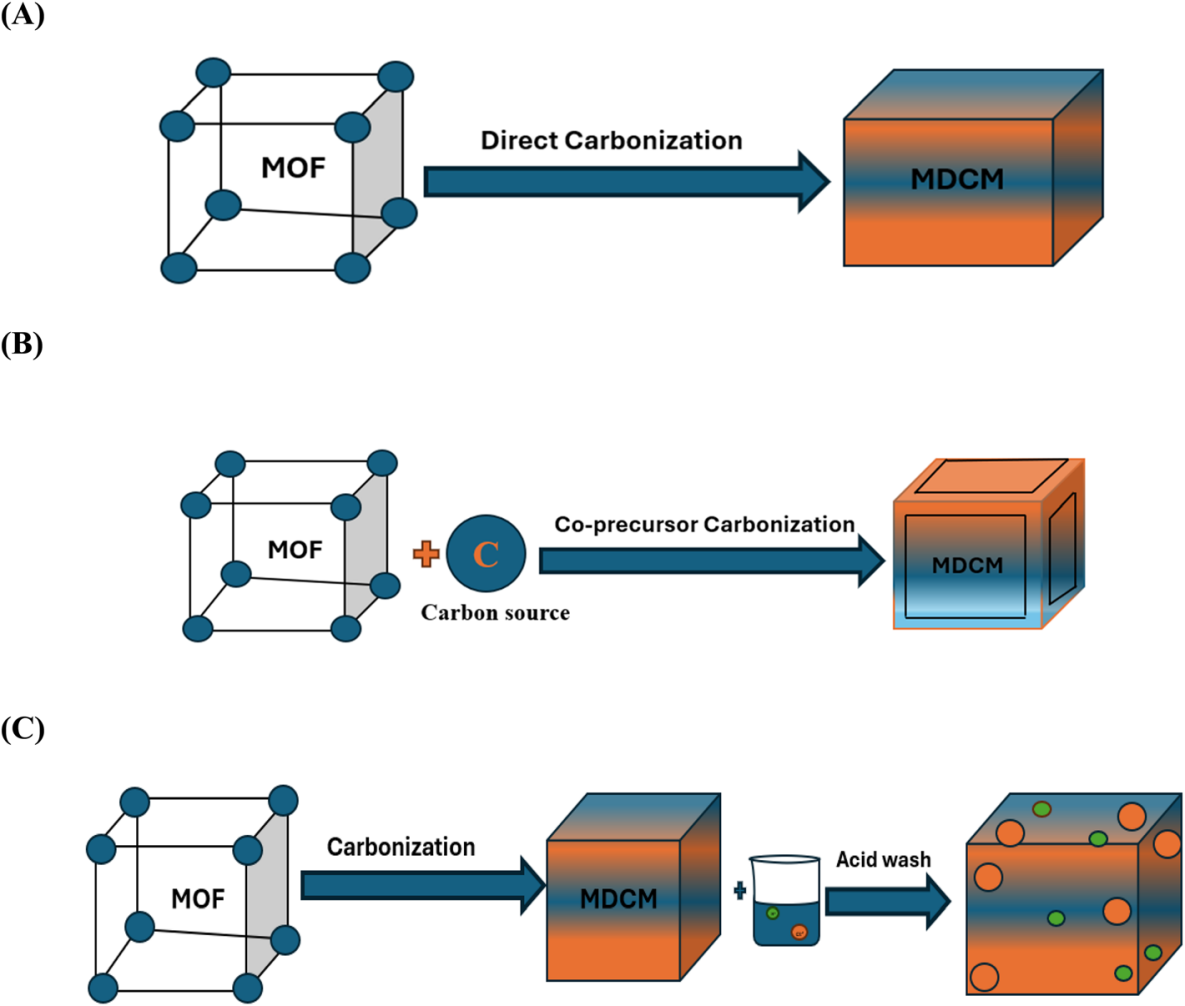
Main carbonization strategies for MOF-derived carbon material: (A) direct carbonization, (B) carbonization of MOF with co-precursor, (C) carbonization of MOF with acid wash.

Three primary methods of MOFs carbonization are available to generate MOF-derived carbons, as seen in Fig. 7: three methods of carbonizing MOFs are (A) direct carbonizations, (b) carbonization with co-precursors, and (C) acid wash carbonization. The characteristics of the carbon compounds generated from MOF are significantly impacted by the preparation approach.^73^ Numerous options for derived carbons are available due to the intrinsic diversity of MOFs. Because MOFs' organic ligands have a high carbon content, they can undergo varying temperatures during pyrolysis to produce metal or nanoporous carbon compounds with distinctive morphologies. Precise management of carbonization conditions can enhance surface area, pore volume, and porous structure while preserving the superior pore volume, porous structure, and performance of MOFs.^79^ The remarkable benefit of being simple to prepare is demonstrated by the synthetic procedure used to create the derived carbons. When comparing the activated carbons created through this method with those sold commercially, they exhibit more organized pore architectures.^80^ As the carbonization reaction process is completed in comparatively less time, this method is more practical, simpler, and faster than alternative carbonization strategies.

### 4.2 Direct carbonization

Numerous investigations have demonstrated that directly carbonizing MOFs is a workable way to prepare carbon. The antecedents of MOFs and the synthesis processes have a major influence on the pore and shape adjustable properties of generated carbons. It is possible to modify and improve the carbon characteristics by adding more metal and organic components to MOFs or by changing their composition. MOF-derived carbons have strong physical and chemical characteristics when compared to carbon generated by conventional techniques.^43^

**Fig. 8:**
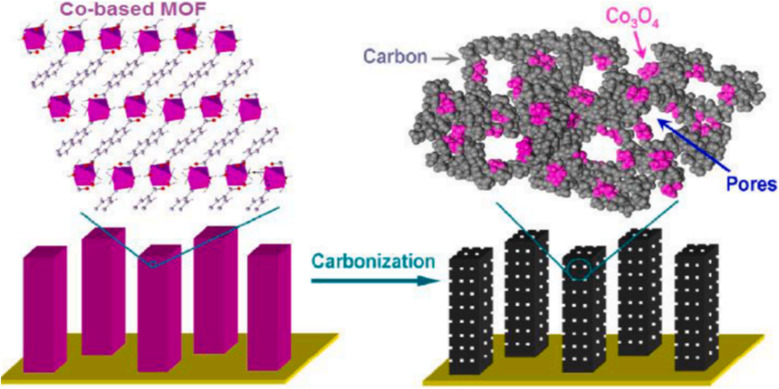
Carbonization process of Co-based MOF into Co_3_O_4_-carbon nanowire arrays.^72^

Ma and colleagues have reported using a cobalt-based MOF as a precursor in a direct, easy, low-temperature carbonisation process at 80 °C in a N_2_ atmosphere to produce hybrid carbon-based nanowire arrays packed with Co_3_O_4_ clusters. As seen in Fig. 8, these black Co_3_O_4_ carbon-based nanowire arrays (Co_3_O_4_-C-NA) were grown directly on copper foil substrate, demonstrating that the Co_3_O_4_ is fully integrated into the carbon species. The Co_3_O_4_ nanoparticles were distributed uniformly and equally.^81^

The Zn-based MOF-5 framework (Zn_4_O(OOCC_6_H_4_COO)_3_) is composed of terephthalic acid (H_2_BDC) and tetrahedral [Zn_4_O]^6+^ units. By annealing, porous ZnO/C composites can be produced. According to Zeng *et al.* (2019), cubic ZnO/C with a surface area of 172 m^2^ g^−1^ was produced by pyrolyzing MOF-5 at 600 °C for three hours.^82^ The generated carbons maintained their cubic shape, and the crystalized MOFs could be rearranged into aggregates. The carbonization procedure caused the organic connectors to undergo pyrolysis, which resulted in the shrinkage of ZnO/C edges and a rough surface.^83^ The interconnectivity of the carbon skeleton can boost material stability while offering an adequate number of response sites.^84^ According to Kukulka *et al.* (2019), MOF-5 was pyrolyzed for two hours at 1000 °C. The carbonized MOF has a considerably greater specific surface area and pore volume than the original MOF (477 m^2^ g^−1^ and 0.33 cm^3^ g^−1^), in 1884 m^2^ g^−1^ and 1.84 cm^3^ g^−1^, respectively. The material's specific surface area and porosity were both increased by carbonization, which also created a porous structure in which the majority of the micropores and mesopores remained.^31^ The synthesis parameters of different MOF-derived porous carbon structures are summarized in Table 1. The same precursor (MOF-5) can be used to create different porous carbon with different surface areas by simply adjusting the pyrolysis temperature, heating rate, and duration.^98,99^ For example, by pyrolyzing MOF-5 in N_2_ atmosphere at 900 °C for 1–2 hours, the derived porous carbon can have a BET surface area above 1500 m^2^ g^−1^. Additionally, pyrolysis of the same MOF-5 precursor with additional precursor furfuryl alcohol can produce porous carbon with a very high BET surface area near 3000 m^2^ g^−1^.^100,101^

**Table 1:** Characteristics of the different porous carbons generated from MOFs

Obtained material	MOF precursor	Additional precursor	Carbonization treatment conditions				Surface area (m^2^ g^−1^)	Ref.
			Temp. [°C]	Gas	Heat rate	Duration		
3D porous carbon	MOF-5	—	900	N_2_	5 °C min^−1^	1 h	1880	82
HPCN	MOF-5	—	900	N_2_	5 °C min^−1^	3 h	1645	84
C-MOF-5	MOF-5	—	900	N_2_	10 °C min^−1^	5 h	1673	85
C-MOF-5	MOF-5	—	900	N_2_	10 °C min^−1^	5 h	1674	86
Mof-DC	MOF-5	—	1000	Ar	5 °C min^−1^	8 h	2714	87
Porous carbon	MOF-5	—	1100	Ar	5 °C min^−1^	8 h	1480	88
NPC	MOF-5	Furfuryl alcohol	1000	Ar	—	8 h	2872	89
NPC_530_	MOF-5	Furfuryl alcohol	530	Ar	—	8 h	3040	90
MC	MOF-5	—	600 900	N_2_	4 °C min^−1^	6 h, 6 h	1812	91
MPC	MOF-5	Phenolic resin	600 900	N_2_	4 °C min^−1^	4 h, 2 h	1543	91
MDC-1	IRMOF-1	—	900	N_2_	5 °C min^−1^	3 h	3174	92
MDC-3	IRMOF-3	—	900	N_2_	5 °C min^−1^	3 h	1678	92
MDC-8	IRMOF-8	—	900	N_2_	5 °C min^−1^	3 h	1978	92
C1000	ZIF-8	Furfuryl alcohol	1000	Ar	—	8 h	3405	93
BF-1000	ZIF-8	Furfuryl alcohol	1000	N_2_	3 °C min^−1^	8 h	1067	94
Nanoporous carbon	ZIF-67	—	800	N_2_	5 °C min^−1^	5 h	—	95
NPC@CNT	MOF-199	CNT	900	N_2_	—	5 h	1370	96
CDM-6	MAF-6	—	800	N_2_	5 °C min^−1^	6 h	1642	97

The original attempt to synthesize nanoporous carbon using the MOF-templated approach was carried out with zinc-based MOF (MOF-5), as reported by Liu *et al.* in 2008. Given its inherent characteristics, MOF-5 is a suitable self-sacrificial template for generating porous carbons.^102^ These characteristics include a high pore volume (1.04 cm^3^ g^−1^) and a high surface area (2900 m^2^ g^−1^), both of which were determined using the Langmuir model. Simple pyrolytic carbonisation of MOF was used by Huang *et al.*, Bakhtiari *et al.*, and Xu *et al.* to successfully synthesize nanoporous carbon.^93^ The production of carbonized nanoparticles (CNPs-T) with ultrahigh surface area and ordered porous structure was reported by Zhao *et al.* using MIL88B-NH_2_ carbonized easily at varying temperatures.^103,104^

### 4.3 Co-precursor carbonization

However, some MOFs have insufficient carbon content because of anisotropic shrinkage or expansion and carbon pore collapse during pyrolysis, which causes larger cracks and a broad pore size range.^105^ This could have an impact on the MOFs' performance in a variety of applications. As an additional source of carbon, MOFs can be used with a variety of different raw materials, such as furfuryl alcohol, ethylenediamine, urea, glucose, ethylene glycol, glycerol, xylitol, and melamine.^87,106^ In comparison with alternative approaches, the co-precursors carbonisation method offers numerous benefits:

(i) The addition of extra carbon sources can raise the particular area of surface and improve electrical conductivity.

(ii) This method can also produce particles with good morphology and a controlled size.

(iii) There is no need for etching or other processes during the process of transformation.^107^

**Fig. 9:**
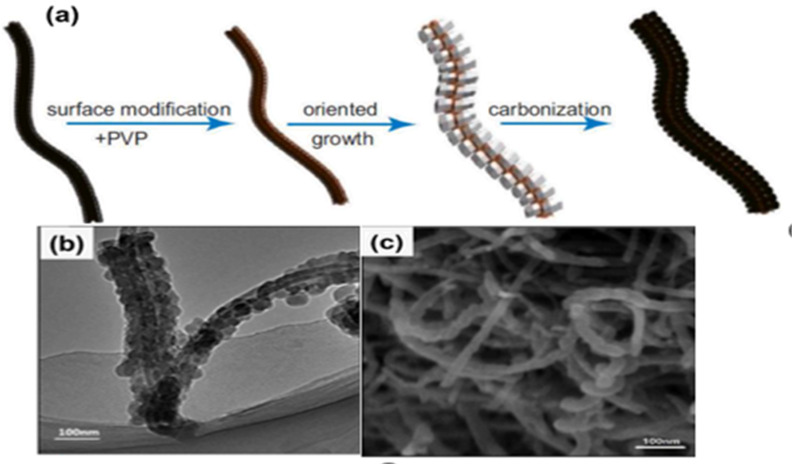
(a) Synthesis method of ZIF-8@MWCNTs necklace-derived carbon, (b) and (c) the TEM and SEM images of the necklace-like ZIF-8@MWCNTs derived carbon.^108^

A ZIF-8@MWCNTs composite was used to create a necklace-like carbon nanomaterial, according to Wang *et al.* The first step in creating the ZIF-8@MWCNTs “necklace” composite was to disperse the MWCNTs in a PVP/methanol solution and then in a 2-methlyi-midazole/methanol solution. After that, the results were gathered and cleaned. Ultimately, as illustrated in Fig. 9a, the porous carbon nanomaterial was created on top of the ZIF8@MWCNTs composite by annealing it for three hours at 800 C while nitrogen gas was present. The ZIF-8 nanocrystals on the MWCNTs (Fig. 9b) were entirely transformed into necklace-like carbon material after the pyrolysis process, and this material was effectively embedded on the surface of the crystalline MWCNTs, as the SEM image in Fig. 9c makes evident.^54^

This special quality and their increased stability over MOFs based on Zr have generated considerable concerns in the real-world uses of Hf-MOFs, especially catalysis.^109^ Long-term durability of hafnium compounds and their gradual reaction rates of conventional uniform solvothermal methods make the discovery for novel Hf-MOFs difficult. UiO-66(Hf),^63^ NU-1000(Hf),^109,110^ MOF-808(Hf)^110–112^ and Corrole-MOF-2 (ref. 113) are a few of the known Hf-MOFs.

### 4.4 MOF carbonization through acid wash

Metal nanoparticles typically aggregate inside the generated carbon compounds during the MOFs pyrolysis process. As a result, the carbon material's pores may become blocked, slowing down the mass transfer process. It is possible to address this problem, though, by using an acid treatment procedure that eliminates superfluous metal nanoparticles, leaving only the metal sites scattered across the MDCM support. In light of this, Yang and associates synthesised a Mn/C–NO nanocomposite by pyrolyzing Mn-based MOF (Mn-BTC) in a N_2_ environment for two hours at 570 °C. After being treated with HCl, the resulting MnO/C powder was annealed for one hour at 900 °C in an NH_3_ environment. The manganese precursor's MnO nanoparticles were successfully eliminated by the HCl treatment, which also made the composite more porous (Table 2). This was demonstrated by the BET surface area (Table 3), which rose following the HCl etching procedure by nearly five times (162.6 to 768.7 m^2^ g^−1^).^114,115^

**Table 2:** Advantages and disadvantages of 3 main types of carbonization process for synthesis of MDCMs^73^

Carbonization method	Carbonization conditions	Advantages	Disadvantages
Direct carbonization	Inert atmosphere	• More affordable and useful	• Not appropriate for every kind of MOF
	Elevated temperature	• Simpler and quicker	• Carbon pore collapse, enlargement, and shrinkage are possible
		• Organized pore structures	
Carbonization with co-precursor	Inert atmosphere	• Higher specific surface area	• More costly
	Elevated temperature	• Enhance electrical conductivity	• Modest response
		• Regulated particle size	
		• Optimal morphology	
Carbonization with acid wash	Inert atmosphere	• Highly porous surface	• Corrosive and unsafe costly
	Elevated temperature	• Clear off any obstructions within the pores	• Conditions for a critical reaction
	Acidic surroundings		

**Table 3:** Comparison of overall merits and demerits of different MDCMs synthesis methods

Synthesis strategy	Methods	Merits	Demerits
Direct pyrolysis	Heating MOF directly in an inert atmosphere (*e.g.*, argon, nitrogen)	Simple, scalable process	May result in low porosity and loss of specific surface area
		High carbon yield	Structural collapse at high temperatures
		Cost effective	Limited functionalization options post-synthesis
Indirect pyrolysis	Pyrolysis with a secondary material (*e.g.*, template or sacrificial agent)	Enhanced control over morphology and porosity	More complex and costly due to additional materials
	Often involves dual steps: carbonization of MOF and removal of secondary material	Produces hierarchical structures	Potential contamination from secondary materials
Carbonization	Heating MOF under controlled conditions (*e.g.*, temperature, atmosphere)	Converts MOF into porous carbon while retaining some structural features	Can lead to shrinkage and structural collapse
	Temperature range: 400–1000 °C depending on desired properties	High surface area and tunable porosity	High energy consumption
		Can achieve high electrical conductivity	Requires precise temperature control to avoid over-carbonization
Template assisted carbonization	MOF is synthesized with a removable template, followed by pyrolysis and template removal	Precise control over pore size and morphology	Requires additional steps for template removal
	Templates include polymers, silica, or other removable materials	Can produce hierarchical and customized pore structures	Risk of contamination from template residues
Direct carbonization of MOF	Direct heating of MOFs in an inert atmosphere	Preserves MOF structure to some extent	Potential for structural collapse
	Commonly used for metal-loaded MOFs	Produces metal-doped carbon composites	Requires high temperatures
Indirect carbonization with precursor doping	MOF carbonization with introduction of dopants (*e.g.*, N, S, P)	Enhances functional properties (*e.g.*, catalytic activity, conductivity)	Complex control over doping levels and uniformity
	Co-pyrolysis of MOF with dopant precursors	Tailored properties for specific applications	Potential loss of MOF structure integrity
Dual stage carbonization	Initial low-temperature carbonization followed by high-temperature treatment	Enhances structural integrity and functionality	Longer process, higher energy consumption
		Produces highly ordered structures	Requires precise control at both stages
Atmosphere controlled carbonization	Carbonization under specific gas atmospheres (*e.g.*, ammonia, CO_2_)	Allows doping and surface modification during carbonization	Requires careful atmosphere control
		Can produce nitrogen-doped or oxygen-functionalized carbons	Additional costs for specialized gases

## 5 MOF-derived multidimensional carbon composites

### 5.1 MOF-derived 2D carbon materials

**Fig. 10:**
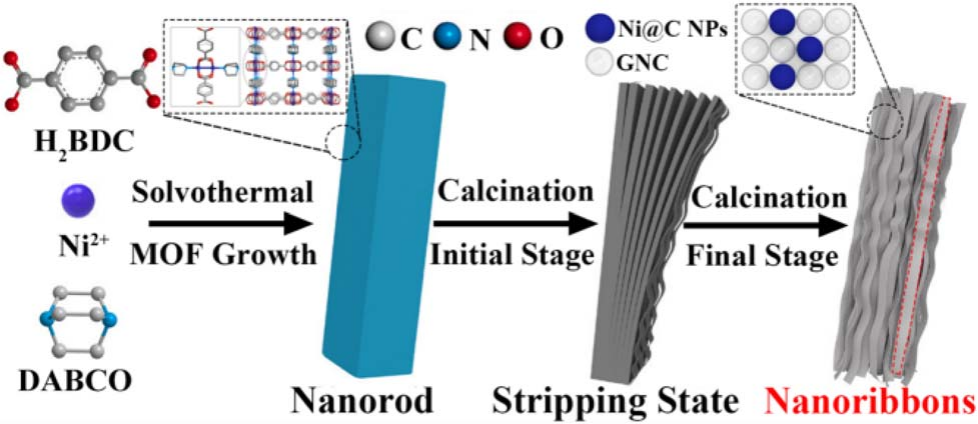
Stepwise synthesis of MOF-derived 2D carbon nanoribbons.^124^

The 2D carbon materials are represented by the “plate” made of few-layer carbon atoms organized in a honeycomb network.^116^ Due to their potentially beneficial qualities, 2D sp^2^ hybridized CNMs have been utilised extensively in the last ten years for electrocatalysis, gas storage, and supporting metal species.^117^ Currently, top-down organic synthesis, chemical or mechanical exfoliation (Table 4), plasma etching, epitaxial growth, and oxidation techniques can all be used to create graphene nanoribbons. Thus, the production of 2D nanoribbons also makes use of porous planar organic crystalline materials as precursors or templates.^118^ Similar to the process of synthesizing 1D CNTs, MOFs of various dimensions can be thermally transformed into well-defined 2D nanostructures by *in situ* or *ex situ* production techniques.^119^ Notably, high aspect ratio, atomically thin 2D graphene nanoribbons exhibit quantum interference effects at the nanoscale, leading to unusual electrical characteristics and greater opportunities in a variety of applications.^120–122^ In contrast to the synthesis of 0D and 1D carbons, the layer-by-layer exfoliation of bulk MOFs is what actually initiates the production of 2D carbon nanoribbons.^123^ Predicted on the synthesis of rod-shaped Ni-MOF rods from nickel nitrate, 1,4-phthalic acid (H_2_BDC), and 1,4-diazabicyclo[2.2.2]octane (DABCO), it is possible to thermally exfoliate the 2D carbon nanoribbon superstructures of graphene nanocages (SGNCs) as shown in Fig. 10.^124^

**Table 4:** Lists the many kinds of MOFs derived carbon composites made for pollutant adsorption and describe the corresponding adsorption processes^213^

MOF derived carbon composites	Pollutants class	Initial concentration	Maximum adsorption	Adsorption mechanism	Ref.
ZIF-8 MDC-1000	Sulfamethoxazole SMX	100 mg L^−1^	435 mg g^−1^	Electrostatic interaction π–π interactions, H bonding	214
MIL-53(Fe)-CNT	Tetracycline hydrochloride (TCN), oxytetracycline hydrochloride (OTC), chlortetracycline hydrochloride (CTC)	200 mg L^−1^	364.37 mg per g TCN, 325.59 mg g^−1^ for OTC, 180.68 mg per g CTC	π–π interactions	215
GO-MIL 101 (Cr)	Indole (IND), quinoline (QUI)	1000 mg L^−1^	542 mg g^−1^ for IND, 498 mg g^−1^ for QUI	π–π complexation, van der Waals forces and hydrogen bonding	216
ZIF-8 CNT	Benzoic acid	1000 mg L^−1^	518 mg g^−1^	π–π stacking interactions	208
Cu-BDC/GrO	Bisphenol	200 mg L^−1^	182 mg g^−1^	π–π interactions and hydrogen bonding	217
Cu-BTC/CNT hybrid composites	Methylene blue	100 mg L^−1^	172 mg g^−1^	π–π interactions	218
Cu-BDC/CNTs	Bisphenol	200 mg L^−1^	164.1 mg g^−1^	π–π interactions and hydrogen bonding	217
NH_2_-MIL-53/wood carbon	Pb^2+^	5–200 mg L^−1^	223.4 mg g^−1^	π–π interactions	219
Ni-BDC/GO	Methylene blue	5–200 mg L^−1^	229 mg g^−1^	π–π interactions	220
ZIF-8 MOF-NC	Cu ions	—	33.44 mg g^−1^	π–π interactions	221
ZrO_2_@rGO	Oxytetracycline	—	198.4 mg g^−1^	Surface complexation, π–π interactions and cation π bonding interaction	222
AC–NH_2_–MIL-101(Cr)	*p*-Nitrophenol	100 mg L^−1^	18.3 mg g^−1^	Hydrogen bonding, π–π interactions, open metal sites Cr^3+^	223
MIL-68 (Al)-GO	TCN	—	228 mg g^−1^	Hydrogen bonding, π–π interactions	224

**Fig. 11:**
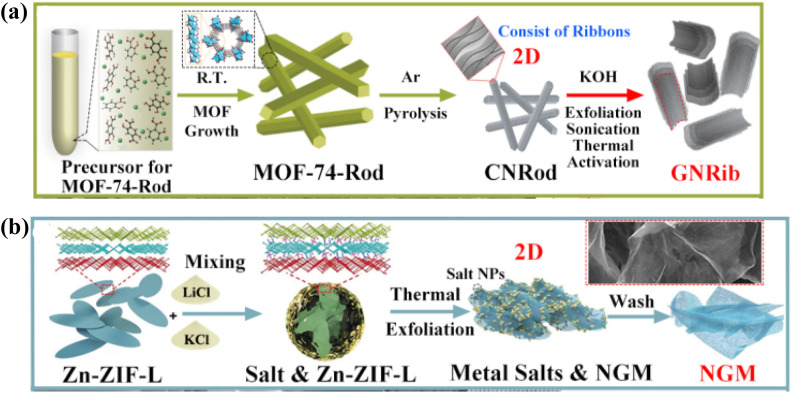
(a) Preparation of MOF-derived 2D carbon nanorods and GNRibs.^72^ (b) Preparation of MOF-derived 2D N-doped graphene nanomeshes.^125^

Zn(ii) sites on the surface of Zn-MOF crystals are stabilized by applying a salicylic acid modulator, as Fig. 11a illustrates, which directs the formation of rod-like Zn-based MOF-74. It has been noted that the zinc species of metals readily volatilize to produce uniform carbon nanorods (CNRods) by pyrolysis. Graphene nanoribbons (GNRibs), which have partially disordered 2D nanosheets and good flexibility and few-layer thickness, are obtained by further treating it with KOH under sonication.^72^

Yamauchi *et al.*, on the other hand, grew different ZIF nanocrystals into nanosheets with a 2D morphology and crystal structure (Zn-ZIF-L).Through the coordination of N atoms and Zn(ii) ions, 2-methylimidazole (2-MI) shows a zigzag chain unit as a bidentate ligand (Fig. 11b), which is further integrated by H-bonding to form a supramolecular framework. Motivated by the distinct layered configuration, the resulting Zn-ZIF-L nanosheets are initially chosen as progenitors rather than traditional ZIFs with three-dimensional structures and they are then exfoliated into ultrathin N-doped graphene nanomeshes (NGMs) by employing alkali chloride as strippers and etchants. The produced NGMs have lots of hierarchical pores, a large specific surface area, high N-doping, and an ultrathin thickness.^125^

Overall, these techniques show how the synthetic approach may be effectively used to generate pure, high-yield 2D *ex situ* formed graphene nanoribbons and nanomeshes on a large scale using different MOF precursors. The 2D CNMs generated from MOFs exhibit favorable performance in metal-ion batteries and supercapacitors due to their advantageous composition and overall structure. Regulators or *ex situ* methods of chemically exfoliating MOF-derived 3D carbon compounds are still necessary for the majority of MOFs in order to achieve dimensional reduction and 2D CNMs.^126^

### 5.2 MOF-derived 3D carbon materials

**Fig. 12:**
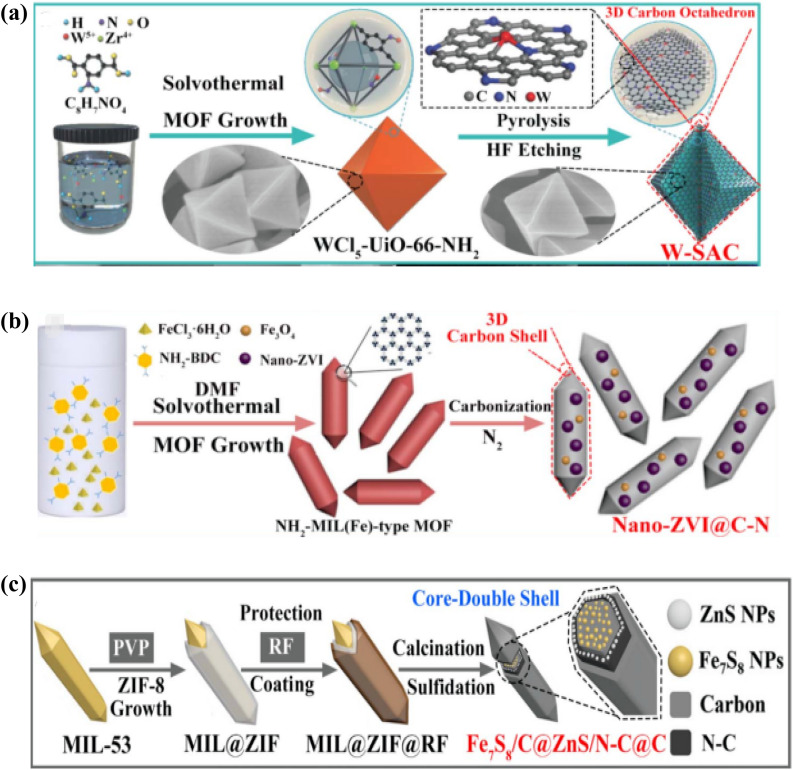
(a) Synthesis of MOF-derived 3D polyhedral W-SAC.^134^ (b) Synthesis of f MOF-derived 3D polyhedral nano-ZVI@C–N.^135^ (c) Preparation of 3D core–shell Fe7S8/C@ZnS/N–C@C.^136^

Currently available 3D carbon materials are primarily made up of typical low-dimensional carbon nanostructures (graphene or CNTs) through self-assembly or aggregation.^127^ By manipulating 3D MOF precursors, researchers have recently directed more of their attention towards material shape, density, and structural orientation. Carbon materials can effectively maintain the morphological features of MOFs during the pyrolysis process because of the comparatively stable coordination network, which makes them an excellent framework support for a variety of applications.^128^ By significantly lowering the material's density, optimizing reaction intermediates, and fostering the carried-out reactions, the resulting 3D porous carbon materials are expected to achieve the desired “low density and thin thickness”.^129^ However, 3D porous carbons are divided into four categories based on their morphology and synthesis method: (1) polyhedral structures made from the original MOFs directly pyrolyzed;^130^ (2) hollow structures resulting from exogenous etching or internal structure collapse;^131^ (3) heterogeneous core–shell structures with a morphological composition that can be adjusted; and (4) other distinct nanostructures like nanoflowers and multi-level hierarchical morphologies.^132^ The ability to maintain their distinct 3D nanostructures from MOF precursors unites them all and directs the manipulation of MOF morphology and composition to produce multifunctional MOF-derived 3D CNMs.^133^ Compared to other kinds, MOF-derived 3D architectures have currently reached a mature state of development in terms of their logical design and simple preparation. As demonstrated in Fig. 12a, where WCl_5_ is first encapsulated within NH_2_-UiO-66, Li *et al.*, for instance, used a pyrolysis method to synthesize a W-based single-atom catalyst (W-SAC). These uncoordinated amine groups hinder the agglomeration of W atoms during calcination. Following that, the final product of W-SAC retains a polyhedral structure, and the excess zirconia may be effectively removed by HF etching, with W and N being uniformly distributed within 3D porous N-doped carbon.^134^

Additionally, as illustrated in Fig. 12b, a spindle-shaped NH_2_-MIL(Fe)-88B is first synthesised and subsequently calcined at a high temperature to yield a 3D hexagonal rod-shaped Nano-ZVI@C–N. Generally speaking, the synthesis of 3D polyhedral carbons by the use of 3D polyhedral precursors has been considered a universal, simple, and efficient method. The idea that the derived active sites are equally distributed over MOF-derived CNMs after high temperature carbonisation is another crucial component of this technique. In these circumstances, the resulting catalysts are able to retain the original polyhedral morphology of their predecessors while also outperforming single low-dimensional structures in terms of stability and performance.^135^

Because of its functional shell, customizable morphology, and highly active core's composition in the inner space, the 3D core–shell nanostructure stands out from other common polyhedral carbons with its plethora of interfaces and significant mechanical strength. Their performance can be effectively enhanced by the careful construction of hierarchical 3D core–shell carbons with distinct layer chemical compositions.^137^

The synthesis of an okra like Fe7S8/C@ZnS/N–C@C compound with a core-double shell structure was described by Sun *et al.* (Fig. 12c). The process begins with the synthesis of a uniform MIL-53 template, which serves as the host MOF for the combination with the guest ZIF-8. The strong affinity of the PVP surfactant modifies the host MIL-53 in this way. This process results in an intriguing MIL@ZIF heterostructure where ZIF-8 particles self-assemble *via* electrostatic attraction. Resorcinol-formaldehyde (RF) is further generated *in situ* on the surface to enhance structural stability. Ultimately, the synthesised MIL@ZIF@RF can be easily converted into core-double shell nanocomposites by calcining with sulphur.^136^

Here, the pyrolytic process is used after the stepwise approach to produce 3D core–shell carbons from prefabricated MOFs. Nonetheless, a thorough examination of the remarkable core-to-shell transformation mechanism during calcination is warranted. Moreover, it should be mentioned that more exact control over synthesis and technology is needed for the building of 3D CNMs with distinct multi-layer shells and heterogeneous cores. It would offer the prerequisites for the simple synthesis of multifunctional 3D nanomaterials generated from MOFs with carefully planned architectures and precisely defined compositions, morphologies, and interfaces.^138^

## 6 Application of MOF derived carbon composite in rechargeables batteries

As the most crucial element in the generation and application of sustainable energy, energy storage is essential to many of our everyday products, including electric cars and smart phones.^139^ Electro materials have been the subject of intense research for the past 20 years, with significant advancements achieved.^140,141^

**Fig. 13:**
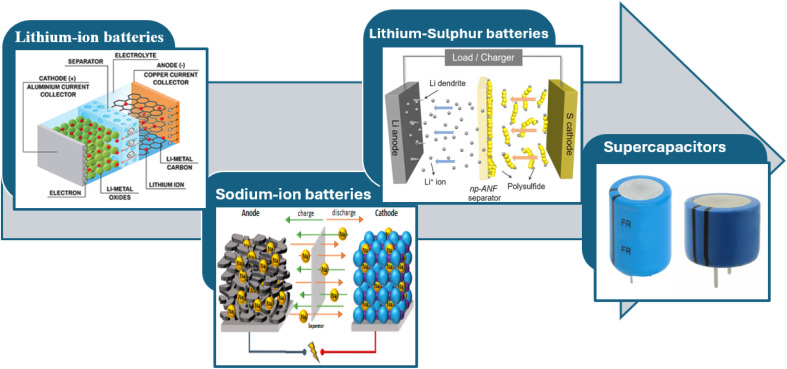
Electrochemical applications of MOF-derived carbon composites.

**Fig. 14:**
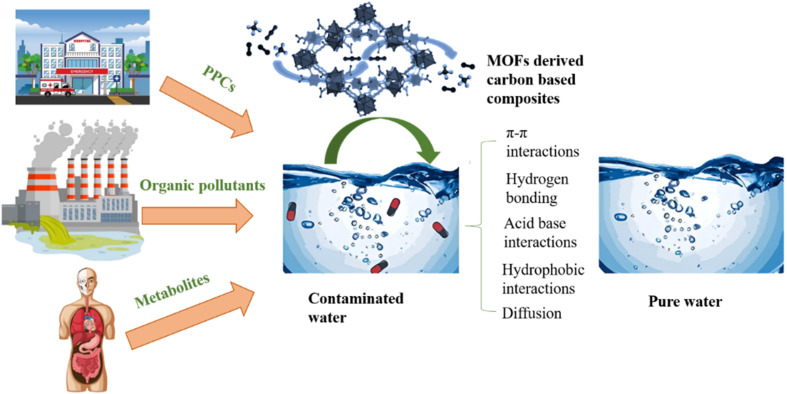
Illustrating sources of pharmaceuticals, organic pollutants and metabolites that are contaminating the water. Removal of contaminants from wastewater by MOFs derived carbon composites and its mechanism.

**Fig. 15:**
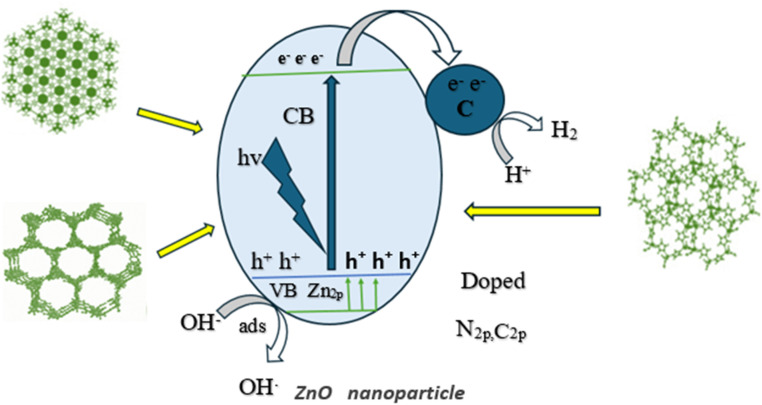
Photocatalytic performance among permeable ZnO/C nanocomposites obtained from MOF-5, MOF-74 and ZIF-8.

The great specific surface area, high electrical conductivity, and superior chemical and thermal stabilities of carbon-based materials have made them popular choices for energy storage applications. The morphology, specific surface area, and size controls of traditional synthetic methods, like pyrolysis of organic molecules or biomass materials, vapor phase decomposition methods, high-temperature solvothermal and hydrothermal methods, and so on, have limitations that make it difficult to explore their reaction mechanisms and fully utilize their electrochemical performance.^142–144^ Furthermore, MOF-derived carbon materials may be modified by deliberate synthetic control in terms of their shape, specific surface area, and particle size, which makes them a competitive type of carbon materials, particularly for energy applications.^145–147^ Finally, the applications of MOF-derived carbon materials in energy storage devices are shown in Fig. 13. In the field of electrochemical energy storage (Fig. 14, 1D carbon-based nanocomposites have demonstrated great promise (Fig. 15). The useful uses and optimization benefits of 1D carbon-based nanocomposites in Li–S batteries, SCs, LIBs, and SIBs will be discussed in this section.

Super-capacitors, a class of high-power density energy storage devices, have drawn more attention than batteries with high energy density because of their superior cycle life, quick charging and discharging times, and high level of safety.^148^

The very low energy density, however, constitutes a major barrier to the extensive use of supercapacitors. Large accessible surface areas, high porosity, good electron transfer capability, and an abundance of redox-active sites are the general qualities of promising electrodes. These strategies have been adopted in order to improve energy densities while maintaining high power densities, based on the energy storage mechanisms of the electric double-layer capacitor (EDLC) and pseudo capacitor.^65,149,150^

### 6.1 Lithium-ion batteries

Secondary batteries are a typical example of environmentally friendly electrochemical energy sources, and they are becoming more and more indispensable in daily life. Presently, LIBs are the most often utilized secondary battery type for energy storage because of their numerous benefits, including low self-discharge, high operating voltage, high energy density, and environmental friendliness. Its further growth is, however, hampered by a few unsolved issues, including unstable structures, dissatisfied energy density and cycling life, and other issues.^151^ Therefore, numerous researchers are currently pursuing the goal of developing electrode materials with a high energy density. Higher theoretical specific capacity materials, like silicon and germanium, have been attempted to replace commercial graphite anode materials. However, their large volume change during the charge–discharge reaction process can easily cause electrodes to peel and pulverize, which is a major hindrance to their commercial applications. On the other hand, materials made of carbon exhibit a solid structure and high electrical conductivity. Micro-nanostructures can have their specific capacity and electrochemical activity increased by carefully building them.^152^ Through a constant redox process involving the transfer of Li^+^ between the positive and negative electrode materials, LIBs may store chemical energy.^41,104,153^ This energy can then be progressively released to power different electronic gadgets and electric vehicles. Many metal oxides have been used as anodes to increase the energy density of LIBs due to their high theoretical capacity. These electrode materials' poor electronic conductivity and slow Li^+^ diffusion paths typically limit their rate performance. Additionally, unstable solid electrolyte interphase (SEI) and volume fluctuations during lithiation/delithiation promote structural disintegration and pulverization, which ultimately results in subpar cycling performance.^154^

### 6.2 LIBs anode

The one-dimensional nanostructure has short Li^+^ diffusion lengths and an effective interfacial contact with the electrolyte.^155^ When used as a LIB electrode, it can effectively prevent substantial volume variation and pulverization/cracking of the electrode materials during cycling. Adding carbon elements to electrode materials is another efficient technique to improve electrical conductivity and reduce volumetric change during the lithiation and delithiation process. The creation and effective use of 1D carbon-based nanocomposites in LIBs offer a wealth of opportunities to fulfil the high power and energy density requirements for the development of next-generation energy storage technologies.^156^ 1D carbon-embedded nanostructures have demonstrated considerable promise for LIBs. These nanostructures comprise of the active component embedded in a 1D conductive carbon framework. Joo *et al.*, for example, described a generic method of adding metal sulphides to CNFs using the tried-and-true electrospinning methodology.^157^ This work involved uniform dispersion of different nanosized metal sulphide particles, including SnS, FeS, Co_9_S_8_, MnS, NiS, and Cu_1.96_S, in CNFs. The composites show strong rate capability and good cyclability when utilized as LIB anodes. Ultra-fine Sn nanoparticles embedded in porous carbon nanorods with N and P codoped (Sn@C) have been used as a LIB anode recently.^158^

Because 1D carbon-coated nanostructures have improved electron and ion transport capabilities, they are being researched extensively. For example, Mai *et al.* reported on pC-SiO_*x*_ NWs, or porous carbon-coated core–shell SiO nanowires, as anode for LIBs. Large volumetric expansion in this work can be mitigated by the 1D structure without cracking during the charge/discharge process. The electrolyte's penetration and subsequent production of a stable SEI film are both aided by the carbon covering layer. As a result, the pC-SiO_*x*_ NWs anode has a capacity of 1060 mA h g^−1^ at 100 mA g^−1^, and it offers consistent cycling stability of over 100 cycles.^159^

Wang *et al.* described a straightforward mechanochemical process in which they formed NPC-clad Si composites (Si@ZIF-8-700N) by wrapping a layer of ZIF-8 *in situ* on the surface of silicon nanoparticles, which were subsequently heated to 700 °C for an hour in an inert atmosphere. Silicon nanoparticles are fully encapsulated by the NPC (ZIF-8-700N) with its porous structure, which buffers the volume change of Si throughout the charge and discharge process. As a result, Si@ZIF-8-700N exhibits outstanding long-life cycle stability and rate performance as a LIB anode material.^160^

### 6.3 LIBs cathode

Qian and Chen *et al.* calcined MOF-74 (Ni) in a reducing atmosphere to produce nanosized Ni particles (5–10 nm) in carbon matrix, and then an *in situ* reaction turned the Ni particles into NiS. C⊃NiS with consistent porosity and tiny particle size (∼50 nm) demonstrated superior electrochemical performance as a LIB cathode as compared to bare NiS. The reversible capacity of the resulting C⊃NiS electrode remained approximately 300 mA h g^−1^ after 100 cycles, whereas the bare NiS degraded to 100 mA h g^−1^ after 20 cycles.^161^

Furthermore, 1D peapod-shaped carbon-encapsulated nanostructures are very promising for improving LIB performance because they have extra internal room to lessen the volumetric shift during charge and discharge. For instance, Zhi *et al.* developed a flexible cathode for LIBs by dispersing V_2_O_5_ nanosheets within carbon nanotubes (V_2_O_5_@G). The nanocomposite as-fabricated exhibits good cyclability, with a capacity decline of only 0.04% per cycle over 200 cycles, and a reversible capacity of 224 mA h g^−1^ at 0.1 C.^162^ Similar to this, Liao *et al.* reported on a carbon-coated Li_3_V_4_(PO_4_)_3_ nanocomposite that was created by high-temperature reactions between LiOH·H_2_O and NH_4_H_2_PO_4_ and a particular V-containing MOFs material (MIL-101(V)). When utilised as the cathode material for LIB, the as-prepared composite material also shown exceptional electrochemical characteristics.^163^

### 6.4 Sodium-ion batteries

The cost of lithium batteries is still very high, which restricts their wider applicability due to the limited deposits and uneven distribution of lithium resources on Earth. Similar to lithium, sodium is abundant, widely dispersed, and relatively inexpensive to extract from the soil. As for SIBs, they are anticipated to be the most promising substitute for LIBs because sodium shares many of the same physical and chemical characteristics as lithium.^164^ SIBs have drawn a lot of attention from researchers because of the inexpensive and abundant nature of sodium. However, slower kinetics, lower energy densities, and a shorter cycle life are caused by the greater radius of Na^+^ (1.02 Å), which restricts the formation of SIBs further. Consequently, numerous initiatives have been made to create Na storage materials for SIBs with large capacities and quick diffusion kinetics.^165,166^ Carbon-based materials have drawn a lot of interest and are frequently used as SIB anode materials. One of the benefits of using 1D C-based composites for enhancing SIB performance is its high level of advantage. The kinetics of Na^+^ diffusion is fast in 1D carbon nanomaterials. Electrical transfer can be efficiently facilitated by their direct pathways. They have the capacity to increase the contact area between the electrolyte and electrode and to give a large specific surface area. Lastly, they may support the strain of Na ions intercalation and deintercalation during the charge and discharge process, which lowers volume variation, prevents active material aggregation and pulverization, and provides long cycle performance.^167,168^

TiO_2_/C nanofibers were established by Yang *et al.* as a SIB anode. This approach prevents the TiO_2_ nanoparticles from aggregating during the cycling process by uniformly embedding them in the CNFs. This electrode demonstrated an impressive 302.4 mA h g^−1^ reversible capacity at 20 mA g^−1^; a high-rate capability of 164.9 mA h g^−1^ at 2 A g^−1^; and a long cycle life of more than 1000 cycles with a nearly 100% capacity retention. SnS_2_ embedded in N and S dual-doped CFRs (SnS_2_/NSDC), which demonstrate enhanced electrochemical performance, was synthesised by Wang *et al.* SnS_2_/NSDC nanofibers work well for Na-storage, demonstrating good electrochemical performance with a rate of 310.6 mA h g^−1^ at 4 A g^−1^ and great cycling performance with 380.1 mA h g^−1^ at 500 mA g^−1^ after 200 cycles.^169,170^ To summarize, 1D carbon-embedded nanostructures have demonstrated notable benefits when used as the anode of SIBs. These benefits include the uniform embedding of ultrafine nanoparticles with reinforced coupling force, easy transport of electrons and sodium ions, prevention of structural degradation, and suppressed aggregation of the active components, all of which improve the SIBs' electrochemical performance. Because of their larger internal void area, 1D carbon-encapsulated nanostructures have been frequently used as anodes for solar-induced battery stacks.

SIBs are recognized as the most potential substitutes for LIBs due to their electrochemical activity, natural abundance, and lack of cost. Nevertheless, insufficient energy density and inadequate cycling stability prevent SIBs from being used in real applications. Because it is more difficult to insert and withdraw reversibly, Na^+^ has a higher ionic radius than Li^+^ (by 55%), which is attributed to its poor cycle life.^70,171^ Thus, it is essential to use appropriate electrode materials in order to further the development of SIBs. However, the absence of suitable active materials for cathodes and anodes has hindered the development of Na-ion batteries.^172^

### 6.5 Lithium–sulphur batteries

Lithium–sulfur batteries have garnered significant interest because of their low cost, high specific capacity, and environmentally beneficial raw material. However, low coulombic efficiency and quick capacity loss are caused by the dissolution of lithium polysulfides (LiPSs) and the insulating property of sulphur.^173^ In contract to LIBs, Li–S batteries are considered the next generation of energy storage systems^174^ because of their high theoretical specific capacity (1675 mA h g^−1^), excellent energy density (2600 W h kg^−1^), and environmental friendliness. However, a few drawbacks still prevent Li–S batteries from being widely used in industry: (1) elemental sulfur's insulating nature S_8_ + 16 Li^+^ → 8 Li_2_S;^153^ (2) the slow kinetics of redox reactions and low utilization of active materials caused by Li_2_S final discharge products; (3) the shuttle effect brought on by the diffusion of soluble lithium polysulfides (LiPSs)^175^ between two electrodes; and (4) the constant volume changes during the charge and discharge process and result in a comparatively low coulombic efficiency.^176^

Additionally, the cathode's structure is negatively impacted by the volume variation that results from the conversion of sulphur to Li_2_S. Fast capacity fading, subpar rate, and poor cycling performance are the results of all these problems. First-generation carbon-based nanocomposites have been used extensively as a possible solution to these challenges in order to advance the development of the Li–S battery.^177^ Firstly, 1D carbon-based nanocomposite with a large specific surface area and good electrical conductivity could be a perfect host for sulphur loading. In the meantime, high aspect ratio 1D carbon-based nanocomposites might successfully prevent the active ingredient from aggregating and promote the use and reaction of sulphur.^178^ Furthermore, because of its superior mechanical properties, the 1D carbon-based nanocomposites could handle the stress resulting from the volume variation during cycling.^179^

Various approaches have been investigated to enhance the performance of Li–S batteries using 1D carbon-based nanocomposites. Adding a porous structure for high sulphur loading is one way to improve the electrochemical performance of 1D carbon-encapsulated nanostructures. Manthiram *et al.*, for instance, described the use of S-a-MCNF, an activated multichannel CNF embedded with sulphur, as a sulphur host material for Li–S batteries. The micropores function as a reaction chamber and the mesoporous multichannel improves the loading and utilization of sulphur, resulting in improved cycling stability of 920 mA h g^−1^ after 300 cycles and good rate capability of 847 mA h g^−1^ at 5 C for the composite cathode.^180^

### 6.6 Super-capacitor

Supercapacitors, another name for electrochemical capacitors, are electrochemical energy storage devices that offer better energy and power densities than traditional dielectric capacitors and batteries, respectively. The extremely long cycle life and quick charge and discharge of SCs are two of its many excellent features.^181^ They might, however, have a lower energy density than batteries. Using several kinds of electrodes can make supercapacitors far more complex devices since composite electrodes can store charge in both capacitive and faradaic ways.^182^

Three categories of supercapacitors can be distinguished based on the various energy storage mechanisms: asymmetric/hybrid supercapacitors, electrical double layer supercapacitors (EDLCs), and pseudo capacitors. Activated carbon is one type of porous carbon that is commonly used as an electrode material in EDLCs. At the electrode–electrolyte interface, ions physically adsorb to provide the source of charge storage in EDLCs. One interesting option for capacitive energy storage is 1D carbon-based nanocomposites due to their enormous surface area. Additionally, 1D porous carbon nanomaterials may keep more active sites visible and prevent nanoscale aggregation.^183^

Pseudo capacitors are devices that store energy from fast-reversible redox processes that take place at the electrode material's surface or near-surface. In contrast to EDLCs, this redox-mediated charge storage method produces high capacitances. Nevertheless, EDLCs have better cycle stability than pseudo capacitors. Applying carbon materials to the pseudocapacitive materials is a useful way to improve the cycling stability of these devices. In recent times, there has been a development of hybrid and asymmetric supercapacitors with the aim of enhancing their energy density even more. An asymmetric supercapacitor is composed of two distinct capacitive electrodes, whereas a hybrid supercapacitor combines a capacitive electrode and a faradaic electrode similar to that of a battery.^184^ High energy density is achieved by increasing capacitance and extending the voltage window by the combination of the various electrodes.

Stretchable supercapacitors that can withstand tensile stresses are gaining more and more attention as a result of the rapid development and use of portable electronic gadgets. A free-standing material for the flexible electrode can be created using a one-dimensional carbon-supported nanocomposite.^185^ For solid-state aqueous asymmetric supercapacitors (ASCs), Yushin *et al.* reported on the growth of an N-doped carbon nanowire/metal oxide nanocomposite directly on conductive carbon fabric. With a broad cell voltage of 1.6 V and a high areal capacitance of 60 mF cm^−2^, the 1D carbon-supported devices offer quick and effective electron and ion routes, improving performance. N-doped carbon nanowire arrays for supercapacitors were published by Zhu *et al.* on carbon nanotube paper (ACNTP/NC).^186^

A new class of energy storage device known as a hybrid SC fills the gap between high-power supercapacitors and high-energy batteries. High-speed batteries, such as metal oxides and sulphides, are used in HSCs to provide high energy density.^187,188^ A porous carbon material with double-layer capacitance is typically used as the opposite electrode to provide high power output. Although HSCs have the potential to attain large power and energy densities, their practical uses are limited by the battery-type electrode's inadequate endurance and slow kinetics. Similar to LIBs, complex nanostructures produced from MOF-based precursors could be used to increase the kinetics and durability of conventional battery-type electrode materials. In alkaline environments, these nano frames demonstrate a high specific capacitance of 2112 F g^−1^ at a current density of 1 A g^−1^ when used as a battery-type electrode material for HSCs. The exceptional cycling performance of NiS nano frames with only 8% capacitance loss over 4000 cycles. Additionally, by expanding the surface area of hollow structured electrode materials with high complexity, the energy storage capacity might be further increased.^189,190^

## 7 Environmental applications of MOF derived carbon-based composites

### 7.1 Pollutants removal

Pollutants originating from diverse sources like industrial wastewater and the combustion of fossil fuels stand out as a pressing environmental issue. Because of its promising potential, efforts have been directed more attention regarding the utilization of MOFs based carbon composites for wastewater purification. Complex organic chemicals found in industrial wastewaters are challenging to eliminate by utilizing standard treatments. Even with the availability of conventional methods like filtration, chemical and membrane technologies, adsorption, sedimentation, and coagulation, reaching the purity standards needed for drinkable water is still a major difficulty. MOFs have unique advantages in wastewater treatment because of their extremely porous structures and adaptable attributes. They improve catalytic activity and adsorption capacity when combined with carbon composites, which makes it easier to remove pollutants from water. These composites work very well in removing heavy metals and organic pollutants, pharmaceuticals from wastewater, which helps to purify it.^46,191^ Concerns about secondary contamination from metal ion leaching are addressed by the higher durability of carbon-based composites derived from MOFs in comparison to MOF precursors or metal ions such as Fe^2+^, Ni^2+^, Mn^2+^ and Co^2+^. Reactive oxygen species are produced under certain conditions to aid in the oxidation of hazardous and persistent organic pollutants into CO_2_, H_2_O or less dangerous byproducts. Porous carbon is highly advantageous for the degradation of pollutants because of its unique structure and performance.^192^

P. Huang *et al.*, used CuO_*x*_-C-550 N MOF derived carbon composite for degradation of ceftazidime from wastewater. Cu-BTC was the MOF precursor. The AO/CuO_*x*_-C-550 N system, showed removal rate 100% for ceftazidime.^193^

J. Wang *et al.*, employed NiO@C composites derived from MOFs for the electrocatalytic degradation of salicylic acid from wastewater. Ni MOF was used as precursor. NiO@C used high performance 99.6% degradation rate for SA.^194^

L. Pun *et al.*, prepared ZIF-8 C_350–400_ (MOF derived C doped ZnO) composite for photocatalytic degradation of phenol and RhB with initial concentration 400 μmol L^−1^ (phenol) and 20 μmol L^−1^ (RhB) from wastewater and observed doping improved the charge separation efficacy and showed high photocatalytic activity in degradation of pollutants from waste water.^195^

B. Niu *et al.*, proposed the ZIF-8 Ag/ZnO@C MOF derived carbon based composites for breakdown of *Escherichia coli* and RhB pollutants with (*E. coli*) concentration 107.0 CFU mL^−1^ and 10 mg L^−1^ (RhB) and instigated their high efficiency 100% for (*E. coli*) and 97.9% (RhB) towards water born bacteria and organic dye in wastewater.^196^

D. Chen *et al.*, suggested Fe–C_500_ composite with Fe-MOF as a precursor for wastewater treatment with a pollutant level 0.36 mM(*i.e.* 4-NP), leading to 89% degradation of 4-nitrophnol.^197^

Tang *et al.*, investigated sulfamethazine was effectively degraded showed 100% efficiency from wastewater with pollutant concentration 20 mg L^−1^ by the FeCu@C composite using H_2_O_2_. It was synthesized using the [Fe, Cu]-BDC precursor and used to effectively catalyze the degradation of (SMT).^198^

W. Shao *et al.*, N-CNTs co-doped with Co/Nx composites were used to degrade bisphenol-A in wastewater, from the pollutant concentration 20 mg L^−1^, and 75.4% efficiency in removal was achieved. ZIF-67@ZIF-8@GO was the MOF precursor that was applied.^199^ S. Yang *et al.*, used CoFe_2_O_4_ NC composites, bisphenol A (BPA) was degraded with an amazing removal efficiency of 97% from an initial 45 μM pollutant concentration. Co/Fe bi-MOF was the precursor used in the composites.^200^

### 7.2 Removal by adsorption

MOFs can be customized to meet particular requirements by modifying elements like pore size, shape, and hydrophobicity by suitable functionalization. The process of tuning entails adding organic functional groups either during the synthesis process or altering already-existing organic linkers or metal sites post-synthesis.^201^ MOFs derived carbon nanocomposites have applications in removal of the pollutants by adsorption from wastewater. When MOF matrices are combined with carbonaceous materials, MOF-carbon composites that are employed in liquid-phase adsorption have improved physio-chemical and functional qualities. The surface properties and porosity of MOF-carbon composites are significantly altered in a linear way. Furthermore, adding functional groups with or without electrons can give the pore surface an electrostatic charge. Furthermore, by cooperating with adsorbate molecules, OMSs inside certain MOF structures can function as chemisorption centers.^202^ A lot of MOFs include open metal coordination sites, flexible frameworks, and different functional groups (such –NH_2_ and –OH) inside organic linkers, which promote powerful interactions like hydrogen bonding and electrostatic interactions.^203^ According to Jhung *et al.*,^204^ adsorbent area of surface plays an indispensable part in eliminating of organic pollutants, especially when the adsorption mechanism only involves van der Waals forces. On the other hand, combining carbon-based materials with MOF components—which have a wide variety of functional groups—improves the association between the sorbent and the targeted, making it easier to apply certain adsorption processes.^192^

### 7.3 Mechanism

Compared to pure components, the synergy of MOFs and carbon composites enhances, revitalize, and magnify their power. High loading capacities are made possible by these materials' totally open periodic porous structures, which are easily accessed by guest molecules. A range of organic contaminants,^205^ such as naphthalene,^206^ pesticides,^19^ tetracycline antibiotics, anti-inflammatory drugs,^207^ and benzoic acid,^208^ are successfully removed using MOF, carbon composites. Different kinds of adsorption methods rely heavily on a variety of interactions. Thus far, a wide range of mechanisms have been identified as being present during the elimination of poisonous contaminants from polluted water through adsorption. The forces like hydrogen bonding, hydrophobic and electrostatic forces, π–π stacking and proton exchange interactions are responsible for elimination through adsorption. In the process of removing poisonous pollutants from water through adsorption, electrostatic interactions are the most commonly seen phenomenon. Surface charge in polar medium, like water, is the electric charge that exists at the contact and disperses. Consequently, water pH level influences the overall surface polarization of MOF carbon composite matrix.^204^ On functionalized MOFs, the adsorption of polar organics is primarily attributed by the mechanism of H-bonding.^71^ For instance, artificial sweeteners adhere by mean of H-bonding during adsorption.^209^ In contrast to other forms of interactions, proton exchange interactions are somewhat uncommon within framework of eliminating organic contaminants from water *via* adsorption. In terms of the impact of metal nodes in the structure, metal cations like Cu^2+^, Zr^4+^, and Cr^3+^ and Fe^3+^, have significant potential as adsorption sites when they act as nodes in the structure. Their capacity to assemble into complexes with the functional groups found in the molecules of organic pollutants gives rise to adsorptive potential.^13,210^ π–π interactions are pivotal in the adsorption of aromatic compounds in aqueous environments for adsorbents based on MOF. Since organic contaminants are naturally endowed with π-electrons, π–π bonds with MOF derived carbon composites formed by these electrons.^211^ These π–π bonds are strongly reinforced by the presence of functional groups on benzene rings, especially in pharmaceuticals. However, it's crucial to remember that the main adsorption mechanism for organic contaminants, including medications, on CNTs is usually π–π Electron Donor–Acceptor (EDA) interactions.^201,212^ Furthermore, hydrophobic interactions are essential for the organic molecules' adsorption from aqueous environments. These substances easily participate in hydrophobic interactions due to their nonpolarity, low water solubility, and extended carbon chains. This behavior is frequently seen when organic materials are being removed from water by adsorption.^204^

## 8 Application of MOF-derived carbon composites in catalysis

### 8.1 Photocatalysis

Photocatalysis has the potential to be used to remove toxins from actual wastewater because it may completely inorganize organic materials and eliminate byproducts. Increasing photocatalytic activity and creating efficient catalysts have been the main goals of numerous studies. Carbons produced from MOFs have been developed as superior catalysts for a variety of photocatalytic processes.^225^ Three Zn containing MOFs such as MOF-5, MOF-74 and ZIF-8 with three ZnO/C nanocomposites with high porosity which was synthesized from three types of MOF-5, MOF-74 and ZIF-8 at submerged vapor condition at high temperature was used to study the photocatalytic process involves the production of hydrogen gas (H_2_) through the hydrogen evolution reaction (HER), as well as the breakdown of organic dyes by photodegradation. From the tendencies observed during the comparison of MOFs and their nanocomposites, it is possible to state that different factors influence the photocatalytic characteristics in various ways. This information revealed that it is possible to achieve the desired properties of the MOF by selection of the correct precursors. By comparing MOF-74, ZIF-8, and MOF-5 ZnO/C photocatalyst with other MOFs, the porous ZnO/C obtained from MOF-5 have the supreme visible light photocatalytic dye degradation efficiency, while the ZnO/C obtained from MOF-74 and ZIF-8 has a higher photocatalytic HER activity up to 9-fold and 4-fold, respectively, than MOF.^226^

MOFs for the last fifteen years has been also considered as one of the major promising sophisticated materials because of the exception in terms of textural characteristics, and great thermal stability and the potential in catalytic processes.^227–231^ MOFs are built up of secondary building units (SBU) that contain metal ions connected to organic linkers by strong coordination bonds, following a process called reticular synthesis, yielding highly porous crystalline frameworks.^232^ For instance, Zn and Ti containing MOFs are anticipated to appropriate for the photodegradation of harmful natural dyes derived from organic sources and function as catalytic agents for HER and oxygen evolution reactions (OER) for H_2_O electrolysis.^233–237^ At the same time, the traditional metal oxide photocatalysts also have severe drawbacks originating from their small surface area, particles aggregation and insufficient operational locations. In an attempt to overcome such concerns, efforts are made towards synthesis of metal-oxide/carbon composites, showing promise for photocatalysts since the band gap of the composites can be controlled. Nevertheless, in the context of physical and mechanical blending, the metal oxides cannot exhibit uniform dissolution within the carbon matrix. Consequently, the lack of effective contact between metal-oxide and carbon leads to a decrease in the photocatalytic efficiency of these composites. Recently, there has been a novel method of incorporating metal-oxide/carbon composites, which are derived from MOFs, directly into carbon matrices. The process entails blending metal oxide nanoparticles with porous carbon networks, ensuring uniform dispersion by subjecting MOFs to temperatures ranging from the initial temperature of the reaction mixture to the temperature at which the metal ions used in MOF synthesis reach their boiling point. By carrying out this procedure in an environment of nitrogen or argon, it is feasible to produce metal oxide/carbon composites with accurate topological structures, various shapes, structures, and capabilities, together with adjustable band gap energies.

**Fig. 16:**
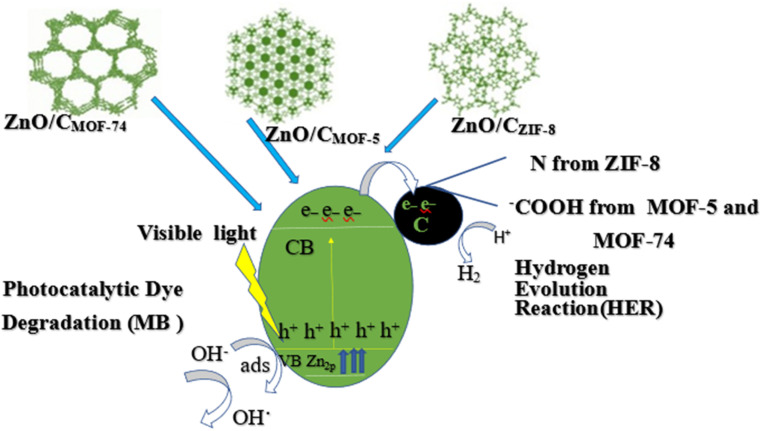
The diagram illustrates the photocatalytic hydrogen evolution reaction (HER) activity of ZnO/C_MOF-5,_ ZnO/C_MOF-74_, and ZnO/C_ZIF-8_, as well as their ability to degrade MB.

These composites including ZnO/C_MOF-5_, ZnO/C_MOF-74_ and ZnO/C_ZIF-8_ were evaluated on the photocatalytic degradation of methylene blue (MB) dye solution and the photocatalytic evolution of hydrogen (H_2_) under visible light conditions to determine their photocatalytic performance. Fig. 16 illustrates the predicted pathway for the breakdown of dyes and the generation of hydrogen (H_2_) through photocatalysis, as inferred from the experimental findings. Therefore, when the energy of visible light matches or exceeds the energy bandgap of ZnO, the visible light energy is used to stimulate the ZnO nanoparticles and generate electron–hole pairs (e^−^/h^+^). The photo-generated holes have the ability to directly oxidize MB, resulting in the formation of neutral species. However, the cavities created by photosensitization can also interact with water molecules (due to the process occurring in H_2_O) and produce (OH˙) radicals. Furthermore, the electrons that are produced during photo excitation in conduction band also engage with molecular oxygen (O_2_) to generate superoxide radical species (˙O_2_^−^). In conventional metal oxide photocatalysts, the conduction band (e_CB_^−^) contains the electrons, while the valence band contains the holes (h_VB_^+^) created by light can readily combine at the catalyst's surface, resulting in significantly poor photocatalytic activity.

Furthermore, metal oxides exhibit elevated surface energies, leading to their agglomeration and subsequent obstruction of the photocatalytic sites. Nevertheless, in composites formed from MOFs, the metal oxides are evenly dispersed throughout porous carbon structure. These composites possess a significant surface area and appropriate pore size, enabling them to absorb a greater number of MB molecules. The MB molecules that have been adsorbed onto the surface of the metal oxide have a greater likelihood of interacting with the photocatalytic active sites. Fig. 16 demonstrates that the electrons within conduction band of the photo can be moved to carbon matrix, causing a delay in the charges of the photo stimulated charge carriers. This delay leads to a reduction in the rate of recombination. The superoxide radicals (˙O_2_^−^) generated from electron-carrying catalysts (e_CB_^−^) react with the adsorbed MB and convert it into benign substances. Within the valence band, the photo-generated h_VB_^+^ and OH˙ radicals undergo oxidation of MB molecules, resulting in their conversion into neutral species.^238^

**Fig. 17:**
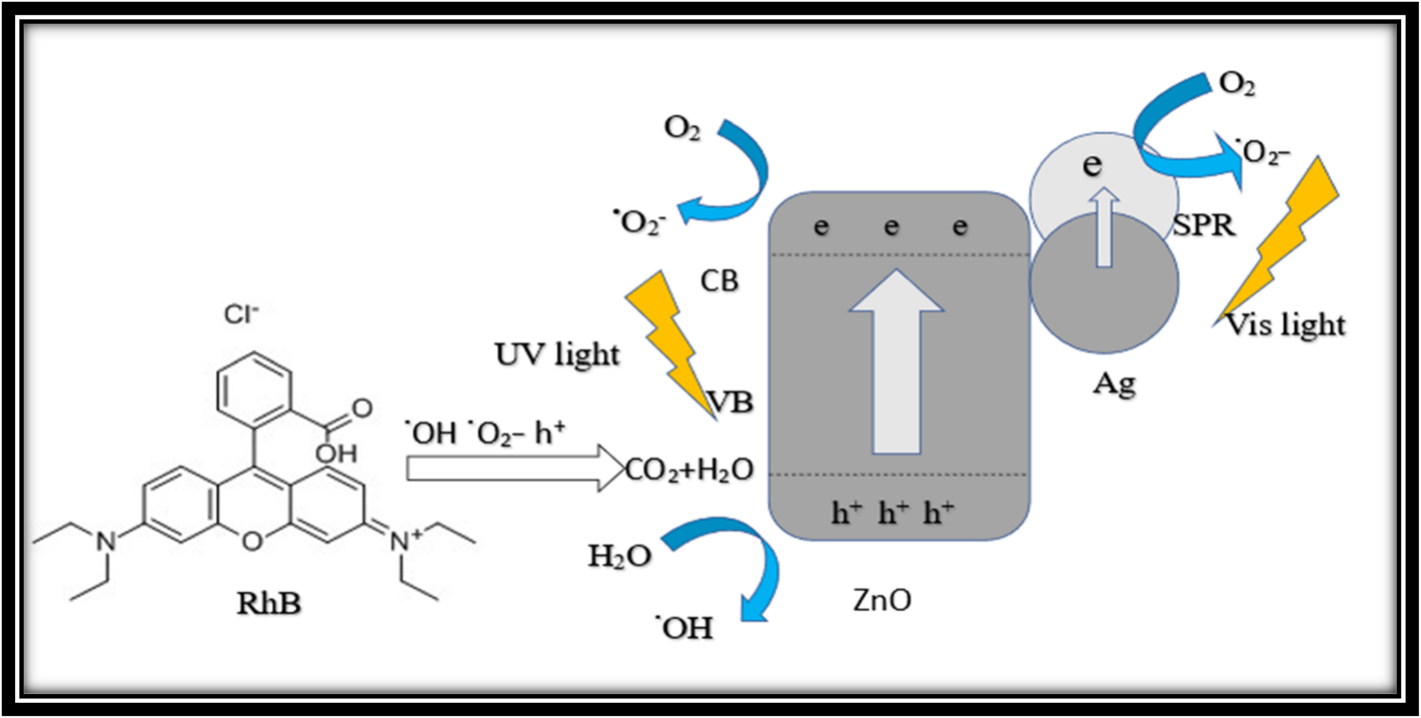
Illustration of photocatalytic process of Ag@ZnO@C.

The process of photocatalytic oxidation, which involves the degradation of MB, occurs *via* participation of ‘holes’ (h_VB_^+^) in the valence band, as well as OH˙ radicals. On the other hand, the reduction process, which leads to the creation of H_2_, is facilitated by ‘electrons’ (e_CB_^−^) in the conduction band. The process of photocatalytic hydrogen evolution (HER) when exposed to visible light was carried out using a solution consisting of 35% methanol (MeOH) in water (H_2_O). The excited electrons in the conduction band interact with the H_2_O/MeOH solution and catalyze the conversion of H^+^ ions into H_2_ gas by reduction. For photocatalytic water splitting to produce H_2_, the semiconductor's conduction band must have a potential lower than H^+^/H_2_O (0 V, NHE), while the valence band must have a potential greater than H_2_O/O_2_ (1.23 eV).^239^ The energy band gaps of ZnO/C composites derived from MOFs vary between 2.9 and 3.1 eV. Furthermore, composites can be readily stimulated by visible light energy, resulting in production of electron–hole pairs. These pairs are crucial for the photocatalytic process of water splitting. Upon exposure to visible light, the ZnO nanoparticles contained within the MOF combine with carbon to create ZnO/C composites, which generate excitons in the form of e_CB_^−^/h_VB_^+^. In this specific context, the electrons are situated in conduction band (CB) and holes are situated in valence band (VB), as depicted in Fig. 17. During the photoexcitation process, electrons are produced, and they occupy the conduction band, whereas the holes are found in the valence band. Subsequently, these electrons and holes experience relaxation processes inside their corresponding bands. The phenomenon of electron separation in conduction band and hole separation in valence band takes place when exciton binding energy is surpassed.

Nevertheless, the dispersion and conveyance of charges to the places where reactions occur are mostly affected by the electronic configuration of the semiconductor. These processes frequently occur at a high speed. Without a doubt, the interfaces between ZnO and C in composites created from MOFs have the potential to improve charge transfer efficiency by reducing recombination. The h_VB_^+^ species, generated during the photochemical process, reacts with MeOH in the presence of OH˙ radicals, which are formed from H_2_O. MeOH is introduced as a scavenger to capture both holes and radicals. This reaction serves to complement the other half-reaction.^240^

Recently, the production of carbon-based nanomaterials from MOFs has resulted in the creation of numerous exceptional photocatalytic materials for environmental protection. ZnO is a significant semiconductor material found in Zn-based MOF-derived carbon. It possesses a high catalytic effect, is cost-effective, environmentally friendly, and has extensive applications in the field of photocatalysis.^241^

A carbon Ag@ZnO@C material was synthesized *via* the carbonization of Ag-doped ZIF-8 at a temperature of 500 °C. This material exhibited highly effective photocatalytic degradation of rhodamine B (RhB) in artificial sunlight conditions. Fig. 17 illustrates that the elevated work function of Ag enables effective transfer of photoexcited electrons from ZnO to the surface of Ag nanoparticles. This reduces the occurrence of the process of separating and combining electric charges. Furthermore, high-energy electrons produced by localized surface plasmon resonance of silver nanoparticles has the capability to catalyze the synthesis of reactive oxygen species (ROS).Nevertheless, following five successive cycles of intermittent photodegradation, the 86th cycle can still yield results, even with a deterioration rate of 3% for RhB.^196^

### 8.2 Organo-catalysis

After creating the porous copper–carbon composite Cu–CC-550 by heat-treating MOF-199 in a single step, it was utilised to decolorize the azo dyes Rhodamine B (RhB) and Methylene blue (MB) in the presence of NaBH_4_.^242^ Strong catalytic activity and good structural integrity were present in Cu–CC-550. Cu_2_O and Cu were the essential elements of catalysis, and they were present in the highly porous amorphous carbon network.^243^ They can transfer electrons from BH_4_ to MB and RhB on the catalyst surface by serving as electron mediators between the dyes (oxidants) and BH_4_ (reductants). The magnetic porous Fe_3_O_4_/carbon octahedra was created using MIL-101(Fe) as the precursor by varying the calcination temperature twice. The catalysts had several mesoporous channels, were composed of graphitic carbons covered with Fe_3_O_4_ nanoparticles, and were widely diffused in water due to the hydrophilic oxygen functional groups on their surface^.^^197^ In just 60 minutes, the Fe_3_O_4_/carbon octahedra demonstrated a strong catalytic reaction that broke down MB with H_2_O_2_. The degradation was about 100%.^244^

Furthermore, Fe-MOF was pyrolyzed to create magnetic carbon nanocomposites (Fe-Cx), and 4-nitrophenol (4-NP) degradation was used to assess the catalytic activity. The catalysts' structure, activity, and composition were all greatly impacted by the annealing temperature. The optimal catalytic performance of the synthesised Fe-C_500_ was seen at a calcination temperature of 500 °C. The rate and efficiency of 4-NP degradation in water were enhanced by the addition of catalyst, H_2_O_2_ dose, and temperature.^245^ While oxidation processes scarcely happened in an alkaline environment, catalytic degradation was supported by acidic circumstances. Fe-C_500_ exhibited good reactivity and catalyzed the 89.0% degradation of 4-NP in 75 minutes. The carbons produced from MOF exhibited great conductivity, porosity, and a high specific surface area. They can enable complete contact with catalysts, oxidants, and pollutants as supporting substrates for nano-catalysts, lessen the agglomeration of nanoparticles, and increase catalytic activity. Carbon catalysts produced from MOFs exhibit remarkable promise for development and application due to their reusability, stability, and low toxicity.^246^

**Fig. 18:**
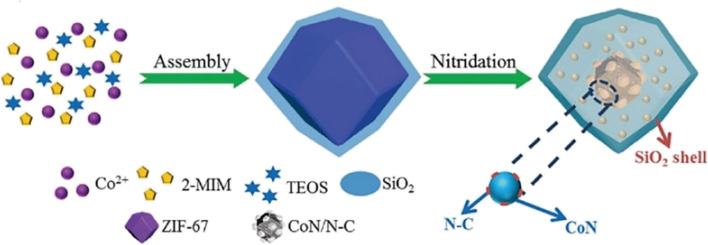
Synthetic procedure of yolk–shell CoN/N–C@SiO_2_ nanomaterials with dual active sites.^79^

The precursor, MOF@SiO_2_, was utilised to create yolk–shell CoN/N–C@SiO_2_ nanomaterials with dual active sites by directly exposing it to the NH_3_ environment as shown in Fig. 18. By triggering PMS, CoN/N–C@SiO_2_ catalyzed the degradation of TC, with a wide pH range of 2.02 to 9.94 and a degradation efficiency of over 95% in less than 30 minutes. In addition to improving catalytic stability, the hydrophilic SiO_2_ shell accelerated the pace of reaction. The complementary roles of free radicals and non-radicals in PMS activation may be realised by the dual active sites of CoN and N-doped carbon as the core. Charges were transferred between Co^2+^, Co^3+^, pyridinic N, and graphitic N, facilitating the catalytic reaction's ongoing advancement.^79^

CoN/N–C@SiO_2_ degrades phenol, BPA, RhB, MB, and MO efficiently. A promising material for aided MOFs production is g-C_3_N_4_, in addition to SiO_2_. In order to produce N-doped porous carbons, boost carbon polarity, and stop pore agglomeration and collapse during pyrolysis, g-C_3_N_4_ can be utilised as nitrogen sources. Furthermore, magnetic co-graphene (MCG) nanocomposites were prepared by Lin *et al.* (2015) using ZIF-67 and GO self-assembly. These nanocomposites were successful catalysts for activating PMS during oxidation. In order to assess MCG's activation power, acid yellow dye was dissolved in water and removed with a about 80% removal rate in 120 minutes. When compared to the carbonized ZIF-67, the electron transport capability in MCG was enhanced by the rGO.^247^

### 8.3 Limitations of MOF-derived carbon nanocomposites in catalysis

Obtaining carbon nanocomposites using MOF typically requires specific temperature and conditions, which makes the process difficult and energy-intensive. It is essential to ensure that metal oxides are evenly distributed throughout the carbon matrix, as any uneven distribution can have negative effects, then there will be inequality in the distribution of the catalytic activity that is displayed by the developed carbon materials. This is true and can be attributed to the fact that the scale up of these nanocomposites from lab scale to large industrial scale is still a challenge because of the need to achieve the same quality and homogeneity as required.^41^ The synthesis of coating process may include several parameters which depends on the substrate and for different samples may also differ in a way that will give non uniform deposition of the coating on the substrate affecting the properties and performance of nanocomposites which is disadvantageous in large scale production. Synthesis of MOF-derived carbon nanocomposites might be challenged by the fact that they degrade when exposed to catalytic reactions.^43^ While MOFs are famous due to thermal stability of the material and, the resulting nanocomposites may not actually be able to withstand high temperatures for a longer duration of time. The above degradations can reduce their potential when used in catalytic applications where high temperature conditions can be maintained. Furthermore, they are not well defined regarding their ability to withstand vigorous reactions such as with concentrated and hot acids/bases and not reacting with oxidizing agents. The first one, if kept for longer durations, deteriorates the nanocomposites' structure and their efficiency as catalysts and using potential, and shortens their life cycle.^248^

Nevertheless, significant efforts in synthesis of MOF derived carbon nanocomposites, problem of charge recombination still persists. The recombinant of the photo generated electron–hole pairs decrease the effectiveness of the photocatalytic reactions. Catalysts with large surface area and porosity are favorable for catalytic reactions, but these measures do not necessarily mean a lot of available active sites. For example, particle agglomeration within the carbon matrix can hinder the exposure of active sites to the reactants and thus lowers the efficiency of the nanocomposites in catalysis. These issues can be partly resolved by enhancing the control over the synthesis and organization of these materials to optimize their catalytic activity.^249^

Another major factor that was raised in relation to MOF-derived nanocomposites is environmental impact and sustainability of the synthesis processes. Some of them require the use of toxic solvents and significant energy thus contributing to sizeable ecological footprints. However, the expense of the starting materials, energy intake and elaborate synthesis process could make these nanocomposites costly compared to conventional catalysts. The economic factors presented here are the reasons why they have not been readily incorporated into industrial uses. Consequently, stoichiometric-kinetically controlled synthesis techniques that can reduce cost and environmental impact are critical to extend the application of advanced materials for catalysis.^250^

### 8.3.1 Future challenges

Because of the large disparities in electronegativity, the bonding interaction between carboxylate oxygen atoms and s-block metal centers is often predominantly ionic. As a result, the coordination geometry is unpredictable and difficult to manage. The spatial relationship of the functional groups has significant implications on the coordinating behavior of the metallic core embedded in structure. Methodologically studying MOFs based on s-block metal ions is highly challenging owing to the erratic coordination behavior and the prevalence of steric effects. Due to limited stability and particularly anticipated chemical properties, s block metal based MOFs have received less attention in both fundamental and applied chemistry.^11^ Because of intricate characteristics of titanium chemistry when dissolved it is difficult to isolate crystalline Ti-MOFs and to achieve regulated assembly of their crystal structures.^16^

One of the key challenges still facing MOF-derived carbon composites is the development of scalable and affordable synthesis techniques. The practical use of current synthesis techniques is limited due to their frequently complex and resource-intensive nature. To make high-quality carbon composites on a larger scale, research should concentrate on optimizing synthesis protocols, such as increasing the efficiency of MOF precursors and creating eco-friendly techniques. Improving the efficiency of MOF-derived carbon composites in rechargeable batteries requires exact control over their morphological and structural characteristics. Better control over pore size, surface area, and doping elements may be possible with advances in materials characterization techniques and a greater comprehension of the production mechanisms, which would increase battery performance. It is crucial to guarantee the long-term stability and robustness of carbon composites generated from MOFs in battery applications. These substances have to endure frequent charge–discharge cycles.^251,252^

For MOF-derived carbon composites to achieve their full potential, their integration with emerging technologies such as flexible electronics, advanced energy storage systems, and next-generation catalytic processes is essential. Researchers are exploring ways to adapt these materials for integration with novel technological platforms and enhance their functionality in these contexts. Recent studies have investigated the compatibility of MOF-derived composites with various emerging technologies and proposed strategies for optimizing their performance.^253^

Although adsorption is useful in combating pharmaceuticals, a number of restrictions prevent the development of better adsorbents. MOF derived carbon-based adsorbent from material like (ACs), (CNTs), and graphene are often commercially restricted due to high manufacturing facilities and regeneration costs. It is still very difficult to dispose of secondary waste, such as recovered pharmaceutical and used adsorbents.^254^

Enhancing the photocatalytic efficiency of the derived MOF-based nanocomposites is another area of research interest. One of the strategies that has been employed in the modification of the nanocomposites electronic structure is bandgap engineering. Improved the content and arrangement, scholars can establish the correct bandgap and photon energy that may bolster photo-absorption and charge separation.^255^ Modification by doping with different elements like noble metals like silver or gold and preparing the alloyed nanocomposites has many advantages: introduction of new active zones and improving the transporting of the charged particles results in the improved photocatalytic performances. Another application of nanocomposites is also being investigated regarding the surface characteristics of the nanocomposites so as to increase competencies between nanocomposites and the reacting materials, making the photocatalytic conversions more efficient.^256^

Thus, the above-discussed MOF-derived nanocomposites are being adopted in environmental catalysis, specifically in wastewater and gaseous phase purification. Owing to their large surface area and the ability to control pore size, mesoporous materials can be effectively utilized for adsorption and degradation of pollutants such as heavy metals, and organic dyes. In water treatment, the application of these nanocomposites has exhibited promising results which include photodegradation of toxic organic dyes that make water sources more purified. Some of the applications of these nanocomposites are in air purification systems in which photocatalytic properties of these materials are used for the degrading of VOCs and other air borne pollutants using visible light which enhances the quality of air inside the buildings.^257^

One of the most recent and innovative applications is the use of MOF-derived nanocomposites within hybrid and composite materials. For instance, it would be possible to develop new materials by blending these nanocomposites with other polymers to improve mechanical characteristics and ease of processing.^258^ These composites can be used in flexible and wearable photocatalytic devices, which extend the ranges of its application. Also, it is possible to achieve better electrical conductivity and carrier mobility of MOFs-derived nanocomposites if combined with graphene or other carbon nanomaterials, which results in enhanced photocatalytic activity. They provide combined characteristics of two components thus making the total performance greater than each of the elements.^259^

Literature review and quantum chemistry simulations are found to be used much frequently in the exploration of MOF-derived nanocomposites. Computational facilities such as density functional theory (DFT) and molecular dynamics are also used to simulate the properties of these materials. These works are helpful for gaining the understanding of the basic concepts of photocatalysis and also the synthesis of new materials.^260^

## 9 Conclusion

Evaluating MOF-derived carbon composites shows that there has been a lot of advancement in their production and use in electrochemical, environmental, and electrocatalytic technologies. As a result of their amazing performance increases in energy storage, environmental cleanup, and catalytic processes, MOF-derived carbons have become a versatile material. Technological developments in synthesis, including template-assisted procedures, controlled pyrolysis, and creative carbonization techniques, have made it possible to precisely tune the structural and functional characteristics of these materials. Improved efficiency and efficacy in a variety of applications have resulted from the ability to customize the pore structure, surface chemistry, and electronic properties of carbon composites formed from MOFs. For the wider use of these materials, the creation of sustainable and scalable synthesis techniques is still essential.

## Data availability

No primary research results, software or code have been included and no new data were generated or analysed as part of this review.

## Conflicts of interest

There are no conflicts to declare.
